# Understanding the sequential activation of Type III and Type VI Secretion Systems in *Salmonella typhimurium* using Boolean modeling

**DOI:** 10.1186/1757-4749-5-28

**Published:** 2013-09-30

**Authors:** Chandrani Das, Anirban Dutta, Hannah Rajasingh, Sharmila S Mande

**Affiliations:** 1 Bio-Sciences R&D Division, TCS Innovation Labs, Tata Consultancy Services Ltd., 54-B, Hadapsar Industrial Estate, Pune 411013, Maharashtra, India; 2 Present address: Novartis Healthcare Pvt. Ltd., #6 Raheja Mindspace, Hitec-city, Hyderabad 500081, India

**Keywords:** *Salmonella typhimurium*, Salmonella pathogenicity island 1 (SPI-1), SPI-2, Type VI Secretion System (T6SS), Boolean modeling, Cross-talk network

## Abstract

**Background:**

Three pathogenicity islands, viz. SPI-1 (Salmonella pathogenicity island 1), SPI-2 (Salmonella pathogenicity island 2) and T6SS (Type VI Secretion System), present in the genome of *Salmonella typhimurium* have been implicated in the virulence of the pathogen. While the regulation of SPI-1 and SPI-2 (both encoding components of the Type III Secretion System - T3SS) are well understood, T6SS regulation is comparatively less studied. Interestingly, inter-connections among the regulatory elements of these three virulence determinants have also been suggested to be essential for successful infection. However, till date, an integrated view of gene regulation involving the regulators of these three secretion systems and their cross-talk is not available.

**Results:**

In the current study, relevant regulatory information available from literature have been integrated into a single Boolean network, which portrays the dynamics of T3SS (SPI-1 and SPI-2) and T6SS mediated virulence. Some additional regulatory interactions involving a two-component system response regulator YfhA have also been predicted and included in the Boolean network. These predictions are aimed at deciphering the effects of osmolarity on T6SS regulation, an aspect that has been suggested in earlier studies, but the mechanism of which was hitherto unknown. Simulation of the regulatory network was able to recreate *in silico* the experimentally observed sequential activation of SPI-1, SPI-2 and T6SS.

**Conclusions:**

The present study integrates relevant gene regulatory data (from literature and our prediction) into a single network, representing the cross-communication between T3SS (SPI-1 and SPI-2) and T6SS. This holistic view of regulatory interactions is expected to improve the current understanding of pathogenesis of *S. typhimurium*.

## Background

*Salmonella enterica* serovar typhimurium is one of the causative agents of gastroenteritis in humans. The infection caused by this pathogen, known as salmonellosis, leads to severe fever, diarrhea and even death in immune-compromised patients. Such infections, although more prevalent in developing countries (with most reported cases in Asia), continue to be a global threat with a significant number of non-typhoid salmonellosis incidences reported from industrialized countries. Unraveling the mechanism of infection by the pathogen has been a major focus for researchers working in the field of enteric diseases. Two Type III Secretion Systems (T3SS) have been primarily implicated to be associated with the virulence of *S. typhimurium*. The genes encoding the T3SS components are located in two pathogenicity islands, namely, SPI-1 and SPI-2 (Salmonella pathogenicity islands 1 and 2), present in the genome of *S. typhimurium*[1,2]. SPI-1 and SPI-2 differ significantly in terms of the types of genes present in them, their regulation, and the environment in which they are expressed. The initial stages of infection by the pathogen are controlled by the genes of SPI-1, which enable the pathogen to invade the epithelial cells of the intestine and induce an inflammatory response [1]. On the other hand, once the pathogen invades the host macrophages and proliferates intra-cellularly, genes corresponding to SPI-2 get expressed [3,4]. Interaction among the major regulators of these two secretion systems (encoded by SPI-1 and SPI-2) have also been shown to play crucial roles in mediating virulence [5,6].

**Figure 1:**
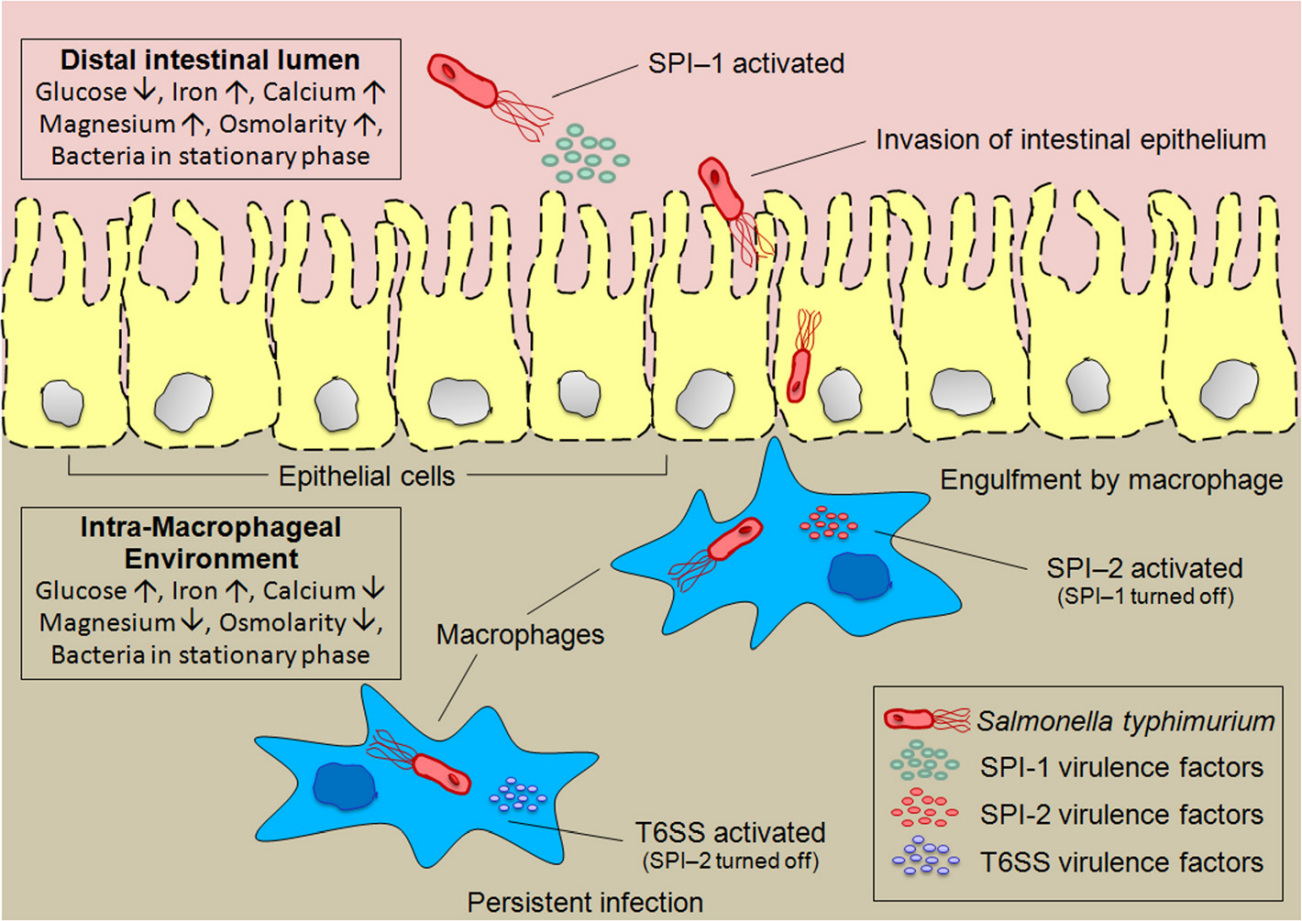
**Salmonella infection.** Sequential activation of different secretion systems of Salmonella during infection. The levels of key environmental factors sensed by the pathogen - first in the distal gut lumen, and subsequently in the intra-macrophageal environment, are mentioned.

In addition to T3SS, the Type VI Secretion System (T6SS) has also been reported to be functionally active in *S. typhimurium*[7]. T6SS has been suggested to play a significant role in later phases of infection. Although the genes constituting T6SS do not participate directly in pathogenesis, they apparently enhance the efficiency of colonization/infection [8]. It has been proposed that, T6SS genes in *S. typhimurium* get expressed in the host macrophage at a late stage of infection when the levels of SPI-2 proteins are reduced [9]. Intriguingly, this inverse regulation between SPI-2 and T6SS has been implicated in helping the pathogen’s persistence inside macrophage by preventing its over-growth and subsequent rapid death of the host cell [9]. In summary, a wide range of experimental evidences suggest that a sequential activation of SPI-1, SPI-2 and T6SS are required during the progression of the chronic infection. A schematic representation of different stages of infection mediated by these secretion systems of the pathogen is depicted in Figure 1. The different environments experienced by the pathogen in course of infection have also been included in the figure.

Previous studies on secretion systems in *S. typhimurium* indicate existence of a tightly controlled gene regulatory network mediating cross-talk among SPI-1, SPI-2 and T6SS [1,4,9]. In order to achieve an opportune and complex regulatory control over the target gene expression, gene regulatory networks are expected to rely on sensing environmental cues using sensor-regulator systems as well as on interactions among different transcription regulators. Although, these two aspects have been widely studied for the network representing interconnections between regulators of SPI-1 and SPI-2 in Salmonella, the cross-talk network between T3SS and T6SS regulators is not yet well characterized. For example, several small-scale network models have been proposed for the gene regulation of the two T3SSs as well as their interconnections [1,5,10-14] mediated through transcription regulators (like HilD) and two-component systems (like PhoP-PhoQ). On the other hand, relatively fewer interactions related to the communication between T3SS and T6SS have been reported. For instance, interaction between two-component systems PhoP-PhoQ (involved in regulation of both SPI-1 and SPI-2) and PmrA-PmrB (involved in regulation of T6SS) have been reported by Soncini and Groisman [15]. An indirect regulation of RcsB (another major regulator of T6SS) by PhoP, mediated via the response regulator MviA, has also been identified in a subsequent study [16]. One of the inner membrane components of T6SS, namely SciS, has also been shown to be negatively regulated by SsrB (transcription regulator involved in SPI-2 activation) [9]. However these interactions, when viewed in isolation, cannot explain the sequential activation of the secretion systems. Unlike for T3SSs, the sensing of environmental signals driving T6SS regulation is not well-defined. For example, although osmolarity has been suspected to have effects on the transcription regulator RcsB [17,18], the exact underlying mechanism has not been elucidated. Therefore, identification as well as integration of the missing interactions and environmental triggers would help in better representation of the cross-talk between T3SS and T6SS, thereby strengthening our understanding of *S. typhimurium* pathogenicity.

The current study focuses on integrating relevant regulatory information associated with SPI-1, SPI-2 and T6SS into a single network and subsequently designing simple mathematical model(s) to gain a systems-level view of gene regulation driving the virulence of *S. typhymurium*. 'Boolean modeling’, particularly useful in cases where there is a scarcity of experimental data [18], has been used in the present study. In Boolean models, each node in a network can attain a binary state, which is either 'one’ (ON) or 'zero’ (OFF), indicating the presence/activation or absence/inactivation of the element represented by the node. At any given time point, the state of each node is determined by the states of its neighbors (interacting with it) at the previous time point. The rule governing the change of state of any node is thus framed depending on the nature of the interactions. In other words, the rules can be logical statements involving the states of corresponding regulatory nodes which are joined using logical operators like 'AND’, 'OR’, 'NOT’, etc. In spite of the simplicity of its logical framework, Boolean models have been successfully employed to study and predict the dynamic behavior of gene regulatory networks [19-25]. Furthermore, the sufficiency of the interactions in a gene regulatory network can be verified through Boolean modeling, based on which novel interactions may be proposed to fill potential gaps [19]. In view of this, a Boolean model of the gene regulatory network of SPI-1, SPI-2 and T6SS of *S. typhymurium* was constructed. An attempt was made to identify previously unreported response regulators (or transcription factors) that may be participating in activation of T6SS. Subsequently, simulation(s) were run with the model in order to verify whether the interactions integrated in this network (including the ones predicted in this study) could mimic the experimentally observed sequential activation of the three secretion systems.

## Results and discussion

### Compilation of relevant regulatory interactions into a Boolean model

The first step towards modeling a gene regulatory network involves compilation of available interaction data from literature. An exhaustive literature survey was performed to identify relevant genes and significant regulatory interactions that are involved in activation of SPI-1, SPI-2 and T6SS of *Salmonella enterica* serovar typhimurium. Since environmental factors (such as osmolarity, metal ion concentrations, availability of sugars, etc.), encountered by the pathogen inside the host during different stages of infection, affect the regulation of these genes, available information about them and their regulatory roles were also compiled. These data provided the foundation for construction of the Boolean model representing the regulation and cross-talk network of Type III and Type VI Secretion Systems in *S. typhimurium*.

**Figure 2:**
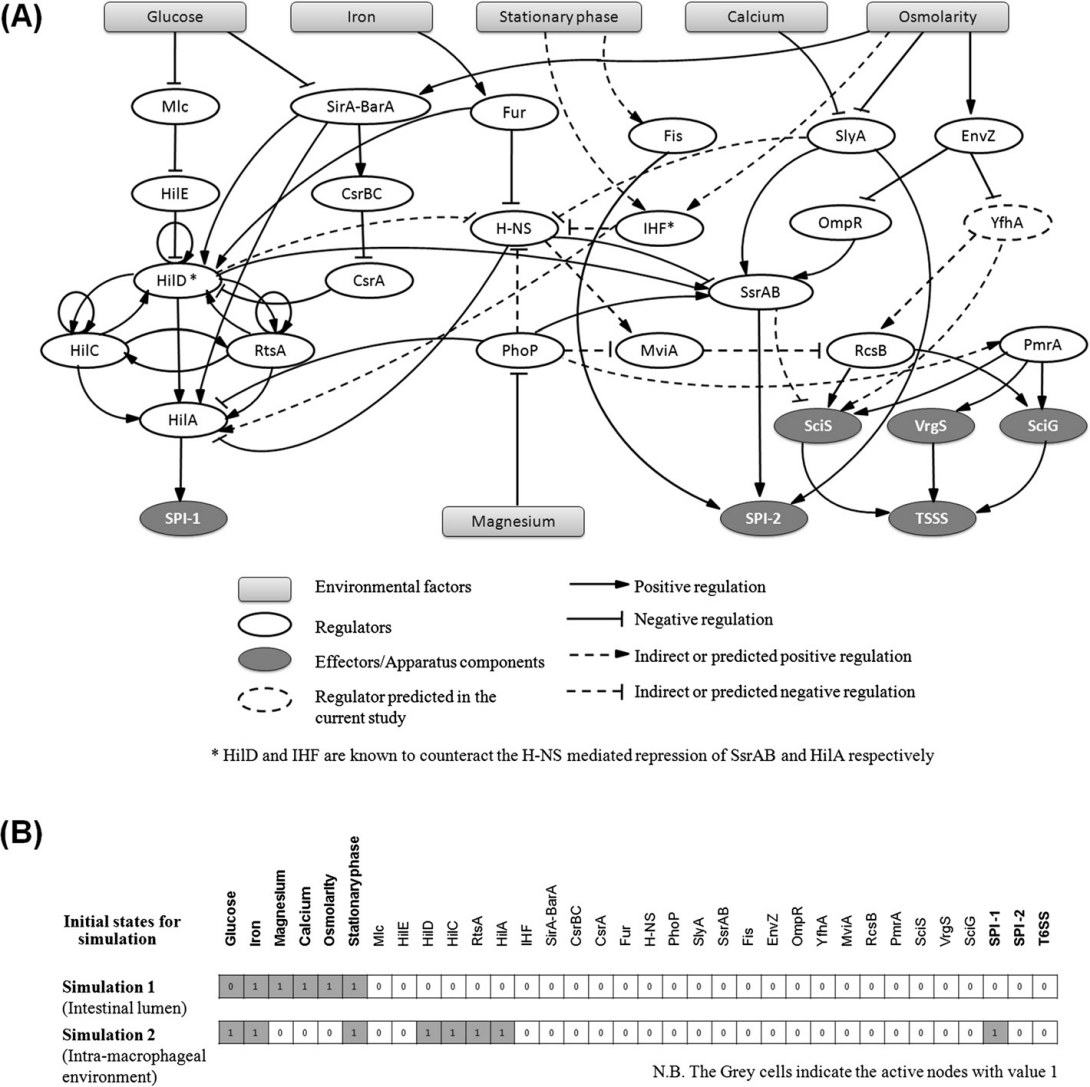
**SPI-1, SPI-2, and T6SS regulatory network. (A)** Constructed gene regulatory network controlling secretion systems of S. *typhimurium*. A Boolean model of this regulatory network was designed by defining appropriate logical rules for the depicted interactions. **(B)** The initial states defined for simulating the Boolean model under two different environments, viz., distal intestinal lumen and the macrophage.

For constructing the Boolean model, the gene regulatory interactions were represented as a 'directed graph’. A schematic diagram of the network is shown in Figure 2. Each node in this graph symbolized either one gene or an ensemble of genes working in tandem (e.g. two-component systems, components of SPI-1, SPI-2 and T6SS). Various environmental factors were also represented by nodes. Each (directed) edge in this graph depicted either a positive or a negative regulatory effect exerted by a particular node on its neighbor or on itself (in case of self-regulation). The genes included in the network are listed in Additional file 1. The gene regulatory cascade leading to virulence is expected to be triggered on receiving certain environmental cues or at certain stages of growth/development of the pathogen. Therefore, except such environmental factors, no 'orphan nodes’ (nodes which lack incoming regulatory connections) were kept in the network. Thus, the network was constructed to closely resemble the *in vivo* regulatory interactions involving the virulence factors of *S. typhimurium* and various environmental factors at the sites of infection inside human body.

### Transcription factor binding sites in the neighborhood of T6SS genes

Since the regulation of T6SS in Salmonella is not well studied, it may be anticipated that a regulatory network constructed with the existing experimental data would lack the expected repertoire of activating factors as well as environmental sensors that are required to trigger T6SS. For example, although previous studies have suggested the role of osmolarity in regulation of one of the principal activators of T6SS, namely RcsB [17,18], the exact mechanism, the sensor-regulator system as well as other intermediates involved in this activation cascade, have not been identified yet in *S. typhimurium*. It was therefore alluring to investigate whether the two regulators of T6SS (viz., RcsB and PmrA) included in the compiled network of interactions can be connected to any additional regulatory cascades sensing the environment, especially osmolarity. The mechanism of osmoregulation of SPI-2 by the two-component system OmpR-EnvZ is well established [26]. Intriguingly, a study on two-component systems in *Eschericia coli* has shown that several sensor kinases have the ability to pair up with multiple non-cognate response regulators [27], thereby facilitating cross-talk between various sensor-regulator pathways. It has also been observed that the osmolarity sensor EnvZ, whose cognate response regulator is OmpR, can additionally phosphorylate a non-cognate response regulator YfhA in *E. coli*. YhfA is normally known to be a cognate response regulator of the YfhK-YfhA two-component system [28], which gets activated during stationary phase of growth (a condition associated with T6SS activation in *S. typhimurium*). BLAST similarity searches indicated that the protein STM2562 of *S. typhimurium* is a close homolog of the *E.coli* YfhA (95% identity, 100% coverage). STM2562 has also been annotated to have a similar function in the NCBI database. Considering that the osmolarity sensor EnvZ is not known to have any other non-cognate response regulator, YfhA was further investigated for its possible role in osmoregulation of T6SS. Since most response regulators control gene expression at the transcription level by binding to the DNA, the neighborhood of T6SS genes were searched for YfhA binding sites. In addition, the Tfsitescan server (http://www.ifti.org/Tfsitescan/) was employed to identify the presence of any other transcription factor binding motifs near the T6SS genes.

#### (A) YfhA binding sites

A search for YfhA binding sites with a previously reported 18 base pair (bp) binding motif [28] revealed three probable hits in the upstream of the *rcsB* gene, when a mismatch of 1 bp was allowed (Additional file 2). Allowing a mismatch upto 2 bp led to the identification of four probable binding sites within the ORF of *rcsB* in addition to multiple hits in the upstream region (Additional file 2).

#### (B) FlrC binding sites

Multiple probable transcription factor binding motifs were detected using the 'Tfsitescan’ server (see 'Methods’) in the upstream of T6SS components (SciS, VrgS and SciG) and their regulators (RscB and PmrA) in *S. typhimurium*. These are listed in Additional file 3. One of the detected binding sites corresponded to the transcription factor FlrC of *Vibrio cholerae*. The upstream regions of *rcsB and sciS* were found to contain copies of a six nucleotide long motif (CGGCAA) identical to the FlrC binding site [29]. Further searches with Tfsitescan resulted in detection of additional FlrC binding sites in the ORFs of *rcsB* and *sciS*. Details of the FlrC binding sites found within the ORFs and upstream regions of *sciS* and *rcsB* are listed in Additional file 4. FlrC has been reported to function in flagellar biogenesis and intestinal colonization of *V. cholerae*[29]. Moreover, the flagellar system has been shown to play a significant role in regulation of T6SS in *V. cholerae* and have also been implicated in the regulation of SPI-1 in *S. typhimurium* in earlier studies [30,31]. A homology search in *S. typhimurium* indicated the protein YfhA to be the closest homolog of FlrC (of *V. cholerae*) with a sequence similarity of 62%, strengthening the possibility of YfhA’s involvement in regulation of T6SS in *S. typhimurium*.

It may be noted here that Tfsitescan could not identify any binding sites for YfhA, probably due to absence of this information in its backend database. Also, only a few of the other binding sites identified could be associated with transcription factors directly involved in virulence related pathways (Additional file 3).

### Structural homology between YfhA of *S. typhimurium* and FlrC of *V. cholerae*

**Figure 3:**
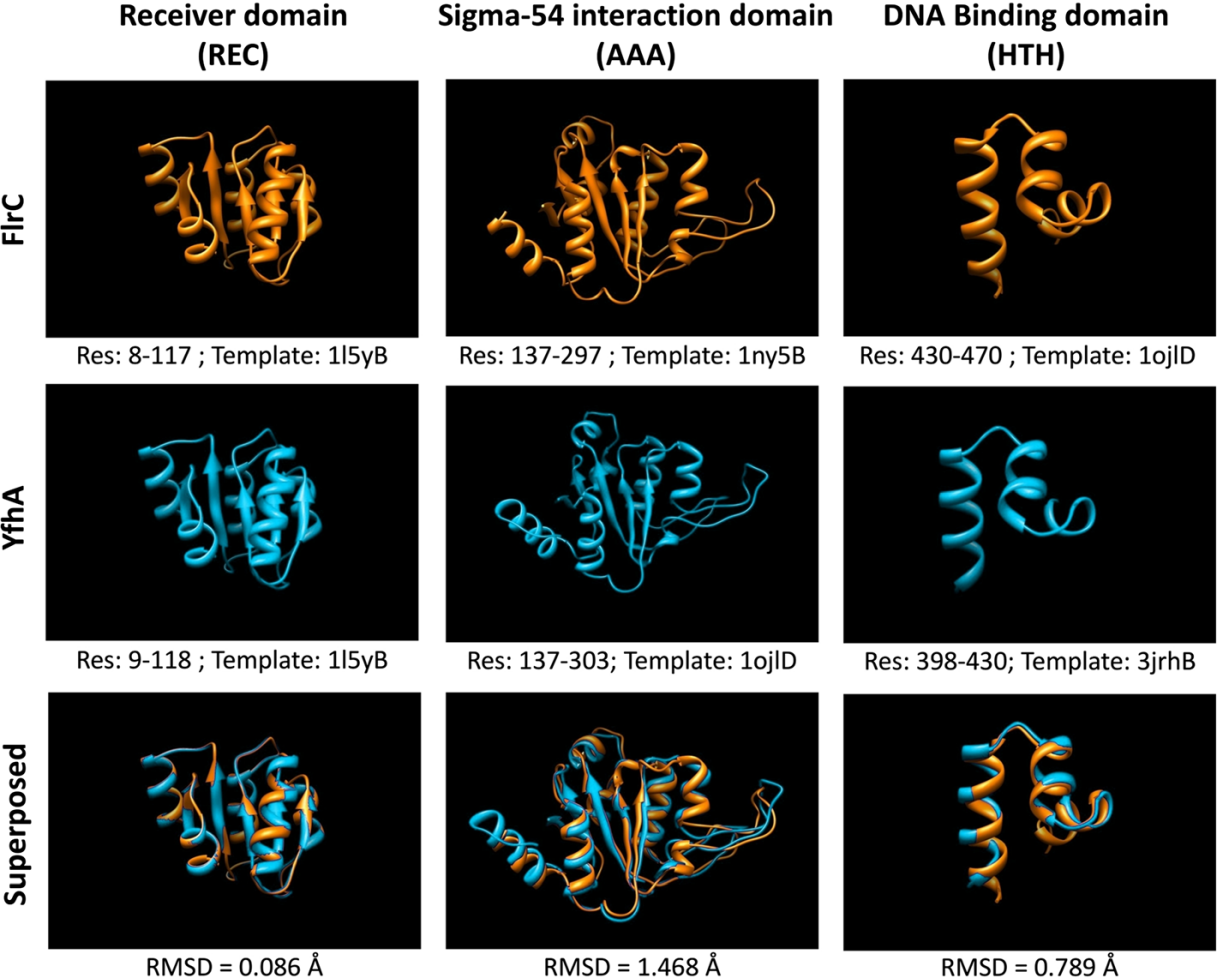
**Structural homology of FlrC and YfhA.** Model structures of REC, AAA, and HTH domains of FlrC and YfhA proteins. The bottom panel shows pair-wise superposition of corresponding domains and their RMSD.

A structural comparison of Vibrio FlrC and Salmonella YfhA (having 62% sequence similarity) was performed to check the extent of their structural homology, which would imply a similar function. Since no crystallographic structures of either of the proteins were available, the comparison was done by building homology models of these two proteins. One of the transcription regulator proteins belonging to the NtrC family from *Aquifex aeolicus* (PDB ID: 1ny5B), having the best matches with each of these two proteins, was considered as the template for building their models. When the modeled structures of FlrC and YfhA were superposed, the RMSD was observed to be 1.32 Å (Additional file 5). A segment-matching approach [32] was subsequently adopted for further evaluating the anticipated structural similarity. FlrC and YfhA are reported as response regulators of two-component systems, and are known to participate in sigma-54 dependent transcription [28,29]. Such response regulators are known to have three distinct functional domains, viz., a response regulator receiver (REC) domain, a sigma-factor interaction (AAA) domain, and a helix-turn-helix DNA binding (HTH) domain. The sequence segments corresponding to these three distinct domains of FlrC and YfhA were identified by searching against the Pfam database [33] (Additional file 6). These domains/segments were then modeled separately using the best available templates (in PDB) corresponding to each of these domains (Additional file 7). Subsequently, pair-wise structural superposition was performed using the models for each of the domains of FlrC and YfhA (Additional file 7). Figure 3 shows the individual and superposed structures of these domains from both the proteins. The RMSD between the respective domains were observed to be 0.09 Å for the REC domains, 0.79 Å for the HTH domains and 1.47 Å for the AAA domains. These results suggest that the YfhA protein in *S. typhimurium* shares significant similarities in structure with the FlrC protein in *V. cholerae*, and may therefore participate in similar functional activities.

### Probable role of YfhA in osmoregulation of T6SS

The presence of several binding sites of YfhA (and its homolog FlrC) in the upstream regions and ORFs of *rcsB* and *sciS*, coupled to the fact that YfhA is also a non-cognate response regulator of EnvZ, strongly indicates the involvement of YfhA in osmoregulation of T6SS. This mechanism is probably similar to that of EnvZ-OmpR mediated osmoregulation of the SsrA-SsrB system [34]. It has been reported that, high osmolarity results in increased phosphorylation of OmpR, a cognate response regulator of EnvZ [34]. In low osmolarity conditions, OmpR and/or OmpR-P (phosphorylayted OmpR present in low levels) can bind to the upstream regions of *ssrA* and *ssrB* genes. OmpR-P, however, have a higher DNA binding affinity as compared to that of OmpR, and when in abundance (as in high osmolarity conditions) can bind to the ORFs of s*srA* and *ssrB*, thereby blocking transcription of these two genes [34]. In the present study, *rcsB* and *sciS* were also predicted to have binding sites of YfhA (and its homolog FlrC) within their ORFs (Additional file 4). Hence, it is likely that there exists a similar mechanism of phosphorylated YfhA (YfhA-P) mediated regulation of *sciS* and *rcsB*. In view of this, it may be hypothesized that, under low levels of osmolarity (typical of an intra-macrophageal environment), unphosphorylated YfhA acts as a sigma-54 dependent transcription factor for the genes *sciS* and *rcsB*, and is responsible for activating the T6SS expression. On the other hand, once the response regulator YfhA gets phosphorylated by EnvZ under high levels of osmolarity, it is likely that it additionally binds to the ORFs of *sciS* and *rcsB*, thus limiting the transcription of these two genes. The missing links connecting osmolarity and RcsB in the Boolean model were imputed based on these assumptions.

### Sequential activation of SPI-1, SPI-2 and T6SS through simulation of Boolean models

Simulation of the Boolean model was performed in order to study the dynamic behavior of the regulatory network and to verify the fitness of the predicted interactions involving YfhA in the model. The Boolean model was simulated under both 'synchronous’ and 'asynchronous’ modes using "GINsim" (http://ginsim.org/home) [35]. The results of the synchronous simulations (state transitions for every node in the network) are provided in Table 1. While simulating the model (allowing synchronous updates) under intestinal environment, the model traversed through seven states before reaching a steady state with SPI-1 turned 'ON’ and other two secretion systems (SPI-2 and T6SS) turned 'OFF’. Subsequently, when the model was simulated under intra-macrophageal environment, it converged to a steady state having T6SS turned 'ON’ and the two T3SSs turned 'OFF’, after traversing five intermediate steps. However, during the second simulation, SPI-2 was observed to get activated and then repressed in two intermediate steps before arriving at the steady state. The results of both the simulations taken together represent a sequential activation of SPI-1, SPI-2 and T6SS, which corroborates well with those observed experimentally [5,9].

**Table 1:** State transitions obtained through Boolean model simulation using 'GINsim’

**(A)**																																		
	**Nodes**																																	
**State Transition**	**Glucose**	**Iron**	**Magnesium**	**Calcium**	**Osmolarity**	**Stationary phase**	Mlc	HilE	HilD	HilC	RtsA	HilA	Ihf	SirA-BarA	CsrBC	CsrA	Fur	H-NS	PhoP	SlyA	SsrAB	Fis	EnvZ	OmpR	YfhA	MviA	RcsB	PmrA	SciS	VrgS	SciG	**SPI -1**	**SPI -2**	**T6SS**
1	0	1	1	1	1	1	0	0	0	0	0	0	0	0	0	0	0	0	0	0	0	0	0	0	0	0	0	0	0	0	0	0	0	0
2	0	1	1	1	1	1	1*	1*	0	0	0	0	1*	1*	0	1*	1*	1*	0	0	0	1*	1*	1*	1*	0	0	0	0	0	0	0	0	0
3	0	1	1	1	1	1	1	0*	0	0	0	0	1	1	1*	1	1	1	0	0	0	1	1	0*	0*	1*	1*	0	0	0	0	0	0	0
4	0	1	1	1	1	1	1	0	0	0	0	0	1	1	1	0*	1	1	0	0	0	1	1	0	0	1	0*	0	0	0	0	0	0	0
5	0	1	1	1	1	1	1	0	1*	0	0	0	1	1	1	0	1	1	0	0	0	1	1	0	0	1	0	0	0	0	0	0	0	0
6	0	1	1	1	1	1	1	0	1	1*	1*	0	1	1	1	0	1	1	0	0	0	1	1	0	0	1	0	0	0	0	0	0	0	0
7	0	1	1	1	1	1	1	0	1	1	1	1*	1	1	1	0	1	1	0	0	0	1	1	0	0	1	0	0	0	0	0	0	0	0
8	0	1	1	1	1	1	1	0	1	1	1	1	1	1	1	0	1	1	0	0	0	1	1	0	0	1	0	0	0	0	0	1*	0	0
**(B)**																																		
	**Nodes**																																	
**State Transition**	**Glucose**	**Iron**	**Magnesium**	**Calcium**	**Osmolarity**	**Stationary phase**	Mlc	HilE	HilD	HilC	RtsA	HilA	Ihf	SirA-BarA	CsrBC	CsrA	Fur	H-NS	PhoP	SlyA	SsrAB	Fis	EnvZ	OmpR	YfhA	MviA	RcsB	PmrA	SciS	VrgS	SciG	**SPI -1**	**SPI -2**	**T6SS**
1	1	1	0	0	0	1	0	0	1	1	1	1	0	0	0	0	0	0	0	0	0	0	0	0	0	0	0	0	0	0	0	1	0	0
2	1	1	0	0	0	1	0	1*	1	1	1	1	0	0	0	1*	1*	1*	1*	1*	0	1*	0	1*	1*	0	0	0	0	0	0	1	0	0
3	1	1	0	0	0	1	0	1	0*	1	1	0*	0	0	0	1	1	1	1	1	1*	1	0	1	1	0	1*	1*	0	0	0	1	0	0
4	1	1	0	0	0	1	0	1	0	1	1	0	0	0	0	1	1	1	1	1	0*	1	0	1	1	0	1	1	0	1*	1*	0*	1*	0
5	1	1	0	0	0	1	0	1	0	1	1	0	0	0	0	1	1	1	1	1	0	1	0	1	1	0	1	1	1*	1	1	0	0*	0
6	1	1	0	0	0	1	0	1	0	1	1	0	0	0	0	1	1	1	1	1	0	1	0	1	1	0	1	1	1	1	1	0	0	1*

The rows represent the dynamic behavior of the network (constituting 34 nodes) under study. ′0′ represents inactive state and ′1′ represents active state. Row 1 represents the values specified as the initial states for simulation. A node’s transit from an inactive state to an active state (0 → 1) or vice-versa (1 → 0) has been marked with asterisks (*). **(A)** Simulation considering the distal intestinal lumen environment, which is the site of infection by *S. typhimurium*. The 8th row corresponds to the steady state attained by the network (representing an activated SPI-1) **(B)** Simulation considering the intra-macrophageal environment faced by *S. typhimurium* after it has been engulfed by the macrophage. The 6th row corresponds to the steady state attained by the network (representing an activated T6SS).

Synchronous simulations, however have a limitation, considering that for the same initial condition, it would always progress through the same path (transition states) leading to the same stable state/cycle. To investigate other possible stable states (and intermediate steps), multiple synchronous simulations were performed wherein only the nodes corresponding to the environmental factors were initialized (fixed) as in earlier simulations. The regulatory nodes for these simulations were set to random initial states (in all possible combinations). Furthermore, simulations were also performed using an asynchronous update scheme. Adopting an asynchronous update scheme makes the outcomes of the simulation stochastic and allows to find out multiple steady states, in case they exist. All these simulations led to the same two steady states, which were identical to the steady states obtained through the synchronous simulations performed earlier (Table 1). The transition probabilities between different states of the system, during asynchronous simulations, were also analysed for the intra-macrophageal environment. The results from the asynchronous simulations (Additional file 8) indicate that the system has a high chance of progressing through paths which correspond to the proposed sequential activation. These results suggest that the Boolean model constructed in the present study fairly captures the *in vivo* regulatory mechanism controlling the cross-talk between T3SS and T6SS in *S. typhimurium*.

An intriguing behavior of the system under study corresponds to the activation of SPI-2 and T6SS under similar environmental conditions inside the macrophages at different stages of the infection cycle. Although, previous studies have suggested a relation between low concentration of SPI-2-secreted proteins and activation of T6SS [9], the mechanism of the regulation is still unclear. From the network constructed in the current study, it can be inferred that several antagonistic connections, such as SsrAB to SciS and MviA to RcsB, may be responsible for keeping T6SS in an inactive state while SPI-2 remains active. Analysis of the state transition table obtained through model simulation shows that, a major inner membrane component of T6SS (SciS) becomes active only when SsrAB is turned off, thus making SPI-2 inactive. SsrAB and SciS are otherwise observed to sense similar environmental signals (like low osmolarity, low levels of magnesium and calcium) through different routes (direct or indirect), which allows activation of both SPI-2 and T6SS in an intra-macrophageal environment. These results suggest that, coordinated activation of SsrAB and SciS, probably plays a crucial role in sequential activation of SPI-2 and T6SS respectively.

To further understand the role of YfhA in the model, a simulation was run under synchronous mode with a (simulated) mutant of this protein, keeping the initial conditions unaltered. The mutation was introduced using the 'mutant simulation’ feature provided in 'GINsim’, which restricted the YfhA node to an inactive (zero) state throughout the entire simulation. Although including this 'mutant’ in the simulation didn’t affect the sequential activation and repression of SPI-1 and SPI-2, the model failed to reach a state with T6SS turned 'ON’ (Additional file 9), indicating the importance of YfhA in the network. It may however be noted that this outcome also relies on the underlying assumption (while defining the logical rules) that sensing osmolarity and magnesium concentrations are equally essential for an opportune T6SS activation.

## Conclusions

A Boolean modeling approach has been utilized in the present study to understand the dynamics of the secretion systems mediating virulence in *S. typhimurium*. In order to unravel the underlying molecular mechanism of virulence of this pathogen, two Salmonella pathogenicity islands (SPI-1 and SPI-2) have been the focus of many earlier studies. Existing literature provides a rich repertoire of gene regulatory information and indicates involvement of a complex cross-talk among various regulatory elements of SPI-1, SPI-2 and T6SS in the pathogen’s virulence. In order to obtain a holistic view of the regulatory interplay, the present study integrates relevant gene regulatory data into a single network, representing the cross-communication between T3SS (SPI-1 and SPI-2) and T6SS. This comprehensive view is expected to improve the current understanding of pathogenesis of *S. typhimurium*. In addition, involvement of new components and interactions has been predicted in an effort to fill in some gaps in our current knowledge of this network. A major challenge towards modeling the cross-talk network under a Boolean framework pertains to properly capturing the intricacies of gene regulation events. This relates to accurately defining the logical rules to ensure the correctness of the model, and to use only the environmental factors (that are critical for triggering any gene regulatory cascade) as input nodes. The logical rules have been defined to maximally reflect the experimentally observed modes of gene regulation involved in the network under study (described in the 'Methods’ section). The environmental factors have been connected to the network in order to mimic different extra- and intra-cellular environments experienced by the pathogen during various stages of infection. Boolean modeling of the cross-talk network and subsequent simulation could reproduce the experimentally observed sequential activation of SPI-1, SPI-2 and T6SS, indicating that, the model could efficiently capture the underlying regulatory mechanism driving the progression of infection *in vivo*.

An interesting outcome of the current study is the identification of YfhA as a potential transcriptional regulator involved in the sub-network corresponding to T6SS. The constructed sub-network for T6SS regulation, based on the information from literature, contains only two regulatory elements (RcsB and PmrA), which have been reported as the major regulators of T6SS genes [36]. Additionally, the sub-network could be connected to only one of the environmental factors (namely, magnesium) through the transcriptional regulator PhoP [37]. Although the suggested role of osmolarity in regulation of RcsB has been reported earlier [17,18], the sensor-regulator cascade remains to be identified experimentally. Imputing YfhA as a missing link would therefore enrich the model, considering that sensing both the environmental factors (low osmolarity, low magnesium) is likely to result in a stronger signal for the T6SS activation cascade, than sensing either one of them. However, such an effect would be difficult to capture through a model based on Boolean-logic, and could probably be better investigated with an ODE-model. Introduction of YfhA in the T6SS sub-network also results in formation of a feed-forward loop involving YfhA, RcsB and SciS. A recent investigation by Le and Kwon (2013) has indicated that such coherent feed-forward loops (FFLs) improve network robustness against update-rule perturbations [38]. Although, validating the effect of this FFL in improving network robustness is outside the scope of this work, the inclusion of YfhA (and the FFL) probably makes the T6SS network better equipped to sense and respond to the environmental cues.

In the current study potential binding sites of YfhA, a two-component system response regulator, has been identified in the upstream region and ORF of the *rscB* gene (one of the major regulators of T6SS). YfhA has also been shown to act as a non-cognate response regulator of the histidine kinase EnvZ [27]. Since EnvZ is a known osmolarity sensor for SPI-2 genes [26], it is likely that YfhA regulates T6SS activation (through RcsB) depending on the osmolarity sensed by EnvZ. Binding motifs recognized by FlrC, a transcriptional regulator found in *V. cholerae*, has also been detected in the upstream regions and within the ORFs of *rcsB* and *sciS* (an inner membrane component of T6SS). Cooperative binding of FlrC near the downstream of transcription start sites of two flagellar genes (*flaA* and *flgK*) has been shown to enhance intestinal colonization of *V. cholerae*[29]. Though FlrC is not found in *S. typhimurium,* analyses performed on the protein sequences as well as their structures suggest that the protein YfhA (from *S. typhimurium*) is a close homolog of FlrC. It may be noted that, the binding sites predicted in this study are based on the motifs recognized by two YfhA homologs from *E. coli* and *V. cholerae*. The motifs recognized by the YfhA of *S. typhimurium* may be different from the ones screened for in this study, which can only be confirmed with further experimental validation. However, these predictions coupled with the information available from literature suggest that inclusion of YfhA in the model is able to bridge the gap pertaining to the anticipated, but hitherto unknown, osmoregulatory cascade controlling T6SS activation. Furthermore, simulation of the Boolean model with a 'mutated’ YfhA did not result in T6SS activation, indicating the probable crucial role of YfhA in the constructed network.

The current study presents a comprehensive model representing cross-talk among the regulatory elements of the three major pathogenicity islands in *S. typhimurum* and predicts probable additional regulatory links to impute certain gaps in the network. Mapping of the dynamic behavior of the cross-talk network suggests the role of coordinated regulation of SsrAB and SciS in successive activation of SPI-2 and T6SS, rather than simultaneous activation. Results obtained in this study are expected to benefit researchers in understanding the sequential activation of secretion systems in *S. typhimurium* and the progression of infection. The integrated view of the gene regulatory network presented here will aid in future studies on *S. typhimurium* infection. Further experimental validation of these findings would strengthen our understanding of *S. typhimurium* pathogenesis. In addition, future efforts towards understanding how host proteins interact with the secreted virulence factors over the course of infection is likely to provide a broader overview.

## Methods

### Search for novel regulatory cascades for T6SS activation

#### (A) Search for YfhA binding sites

A previous study on *E. coli* had indicated a 18 bp motif recognized by YfhA [28]. Multiple copies of this motif [TGTCN(10)GACA] has been shown to be present upstream of the *glmY* gene in *E. coli* which is regulated by YfhA. Presence of this motif was searched in the upstream regions of the major components of T6SS, namely, s*ciS*, v*rgS*, s*ciG*, as well as the direct regulators of T6SS components, namely, *rcsB* and *pmrA*. In cases where a gene was preceded by other genes in its respective transcription unit (operon), the search was performed in the upstream regions of the corresponding operon. Information about putative transcription units were retrieved from DOOR (Database of prokaryotic operons) [39].

#### (B) Search for other transcription factor binding sites

The upstream regions of the T6SS components and their direct regulators were also screened for the presence of other Transcription Factor (TF) binding sites. The tool "Tfsitescan" (http://www.ifti.org/Tfsitescan/) [40] was used to detect such TF binding sites. Subsequently, the list of transcription factors reported by "Tfsitescan" were filtered based on their functions (such as their association to virulence) in order to narrow down on probable regulatory candidates which could also have connections to the anticipated EnvZ-YfhA mediated osmolarity sensing.

#### (C) Structural study of YfhA and FlrC

The search for TF-binding sites indicated several occurrences of the TF-binding motif corresponding to one of the flagellar regulatory proteins (FlrC) of *V. cholerae* (see 'Results and Discussion’). Sequence similarity searches also asserted FlrC to be the closest homolog of the response regulator YfhA from *S. typhimurium*. The extent of structural relatedness (implying a functional similarity) between these homologs was compared using a homology modeling approach. The model structures were generated with the help of Swiss Model server [41] by providing appropriate template structures (see 'Results and Discussion’). For checking the structural similarity, the homology models were superposed with the help of the freely available software 'FAST Alignment and Search Tool’ [42] and RMSD value(s) were noted.

### Boolean model construction

The network of interactions was modeled under Boolean/logical framework using the tool 'GINsim’ (http://ginsim.org/home), which provides a user-friendly interface for simulation and efficient analysis of regulatory graphs [35]. Model building using GINsim involved the following three steps, namely, (i) construction of a regulatory network consisting of nodes and edges, (ii) defining logical rules for each node, and (iii) defining basal and maximum levels of expression for each node. The network was constructed using the experimentally verified interactions obtained from literature. In addition, the predicted regulatory cascade involving YfhA (see 'Results and Discussion’), was also included. The network contained 34 nodes representing genes, their regulators, as well as environmental factors, and 65 edges representing positive or negative regulations. The logical rules for each node were defined based on the modes of regulation of the node by one or more regulators. The logical rules and corresponding references to literature are provided in Additional file 10. In case of a node having multiple regulators (positive and/or negative), 'AND’ or 'OR’ operators were used in the rule based on the relative effects of the regulators on the activation/repression of the particular node. For example, HilA, the main regulator directly activating SPI-1 genes, has been reported to get sufficiently expressed in the presence of HilD along with HilC or RtsA [11]. Hence the rule defined for HilA was "HilD AND (HilC OR RtsA)". For cases, where adequate information regarding the relative effects of the regulators were not available, the 'OR’ logic was applied. One such case was observed for HilD, a major transcriptional regulator involved in expression of SPI-1 genes. In this case, HilD was subjected to positive regulation by four nodes (SirA-BirA, HilC, RtsA and Fur) along with self activation [11,14,43,44]. Negative regulations were included in the rules (wherever reported) with an 'AND NOT’ operator, since all negative regulations were reported to be significantly repressive (except in case of H-NS). The rule defined for H-NS, a gene involved in nucleoid organization, differed from this standard assumption. H-NS was observed to have five negative regulators (HilD, PhoP, SlyA, Fur and IHF), either direct or indirect, relative effects of which were not available from literature. While H-NS has been reported to act as a repressor for a wide range of genes, H-NS mediated repressions have been shown to be counter-acted by different regulators. Such examples include counter-effect of HilD on H-NS mediated silencing of *ssrA* and *ssrB*[5] and counter-effect of IHF on H-NS mediated repression of *hilA*[45]. To account for such multi-factorial effects, the rule for H-NS was defined such that H-NS was turned 'OFF’ only in the presence of all five repressors (HilD, PhoP, SlyA, IHF and Fur). In other cases wherein one or more of the repressors of H-NS were absent, the rules were appropriately defined to consider the counter-effects on H-NS mediated repression (without turning 'OFF’ the H-NS node itself).

The basal and maximum levels for the nodes corresponding to the environmental factors were defined based on the information obtained from the existing literature. The reported availability of these factors in the distal intestinal lumen (site of initial stage of infection) and in macrophages (site of late stages of infection) infected with *S. typhimurium* were considered for the model. Glucose has been shown to mostly get absorbed in the small intestine, limiting its availability in the distal intestine [46]. On the other hand, inside the macrophage, the pathogen has been reported to require glucose for causing successful infection [47]. Osmolarity, magnesium and calcium have been found to be significantly higher in distal intestine as compared to those in the macrophage [14,48-50]. Thus to accommodate for the changes in environment, the basal and maximum levels were kept as 'zero’ and 'one’ respectively for 'Glucose’, 'Osmolarity’ 'Magnesium’ and 'Calcium’. The concentration of iron has been shown to remain comparatively high in the intestine due to absorption of dietary iron [14]. Also during later stages of infection, high iron concentration has been reported to aid in bacterial survival and multiplication inside the macrophage [37]. Thus, to account for high iron concentration both during initial (inside distal intestine) and later phases (inside macrophage) of infection, the basal and maximum values for 'Iron’ were defined as 'one’. Similar values were assigned for the node 'Stationary phase’, as, under oxygen-limiting condition (the site of pathogenic colonization inside human body), *S. typhimurium* in stationary phase has been shown to exhibit optimal invasiveness [51,52]. Among other nodes, the basal and maximum levels of the nodes having at least one positive incoming connection (e.g., HilD, Fur, RcsB, etc.) were defined as 'zero’ and 'one’, respectively. For the nodes having only negative incoming connections (e.g., HilE, CsrA, H-NS, etc.), both the levels were made as 'one’ assuming that, they are constitutively expressed in absence of any repressors. The Boolean model was thus constructed by integrating all the relevant experimental observations. It may however be noted that the environmental factors considered in the current study only provides a simplistic view of the *in-vivo* conditions encountered by the pathogen. There can be several other factors associated with the virulence mechanism. For example, phagocytic free radicals (like hydroxyl radical HO∙) have been reported to cause DNA damage. In response to such DNA damage, intra-cellular Salmonella has been reported to induce SOS response, which mainly includes differential expression of genes like *recA*, *uvrABY*, *lexA*, *sulA, sodA, sodB,* etc. [53,54]. Previous studies have also suggested an SPI-2 dependent mechanism in *S. typhimurium* which inhibits the production of reactive oxygen species [55]. However, no reported regulatory interaction(s) could be found (from existing literature) which indicate any direct role of SOS response (or other such factors) in activating any of the three secretion systems considered in this study. As more experimental data gets available in future, it should be possible to incorporate several such environmental factors and response mechanisms to extend the proposed model.

### Simulation of the Boolean model

The Boolean model was simulated with GINsim [35], in order to draw inferences about the dynamic behavior as well as the steady states of the system. Simulations were performed using both 'synchronous’ and 'asynchronous’ modes. 'Synchronous’ mode offers a deterministic update schema, wherein each node is updated once in a single time step, leading to change in the network state at the end of each update cycle [56]. Adopting an 'asynchronous’ update scheme allows updation of any randomly selected node at a time step, thereby making the outcomes stochastic. While performing the simulation runs, only the environmental factors were considered as input nodes. The environmental factors included in the current study were osmolarity, stationary phase of growth as well as availability of glucose, magnesium, calcium and iron.

Activation of SPI-1 is known to lead to the invasion of intestinal epithelial cells by *S. typhimurium*, and subsequent engulfment of the pathogen by macrophages. Thus, in order to reflect this change in environment experienced by the pathogen, the model under study was simulated with two different sets of initial conditions. These conditions corresponded to the environments in the distal intestinal lumen and in the macrophage. While simulating the model for the distal intestinal lumen environment, the initial state for 'Glucose’ was set at 'zero’, and other environmental factors were set at 'one’, according to their expected availability at the beginning of the infection cycle (described in the previous section). For all other nodes, various possible combinations of 'zero’ and 'one’ were considered as initial states for different rounds of simulation, to witness the behavior of the system constrained only by the environmental cues and the defined logical rules. Similarly, while simulating the model for intra-macrophageal conditions, the values for the environmental factors 'Glucose’, 'Iron’ and 'Stationary Phase’ were set as 'one’, whereas 'Magnesium’, 'Calcium’ and 'Osmolarity’ were set to 'zero’. It was also assumed that in the immediate aftermath of engulfment by the macrophage, the SPI-1 secretion system of *S. typhimurium* and its main regulators (viz., HilD, HilC, RtsA and HilA) would still be in an active state. Thus, the state of these nodes were set to 'one’ in the model corresponding to the intra-macrophageal environment. In summary, the initial conditions for the simulation were defined in order to reflect the *in vivo* state of human distal intestine and macrophage during the infection by *S. typhimurium*.

## Competing interests

The authors declare that they have no competing interests.

## Authors’ contributions

SSM and HR conceived the idea and design of the methodology. HR carried out initial analyses on a smaller Boolean network. CD, AD and SSM generated a comprehensive regulatory network based on the available literature. CD and AD carried out detailed analysis and simulation of the Boolean network. CD, AD, and SSM analysed all the results and drafted the manuscript. All authors read and approved the manuscript.

## Supplementary Material

Additional file 1 **Components of the gene regulatory network.** List of the genes (and RNAs) included in the network representing the cross-talk among regulatory elements of Type III and Type VI Secretion Systems in *Salmonella typhimurium*. The synonym codes correspond to the strain *S. typhimurium* LT2. Click here for file

Additional file 2 **YfhA binding sites.** YfhA binding sites found in the upstream region and in the ORF of *rcsB*. YfhA is known to recognize an 18 base pair long motif [TGTCN(10)GACA]. The results given below indicate the position of the probable binding sites (with respect to the transcription start site), and the number of mismatches allowed while considering a hit. Click here for file

Additional file 3 **Transcription factor binding sites predicted by Tfsitescan.** Transcription factor binding sites predicted by "Tfsitescan" in the upstream regions of Type VI Secretion System genes (*sciS*, *vrgS* and *sciG*) and the genes of their regulators (*rcsB* and *pmrA*). Click here for file

Additional file 4 **FlrC binding sites.** FlrC-binding sites predicted through 'Tfsitescan’ in the upstream regions and within the ORFs of *sciS* and *rcsB*. Click here for file

Additional file 5 **Homology models of FlrC and YfhA.** Details pertaining to homology modeling of the proteins FlrC and YfhA and results of superposition of the two modelled structures. Click here for file

Additional file 6 Pfam Domains identified in FlrC and YfhA. Click here for file

Additional file 7 **Homology modeling of three domains of FlrC and YfhA.** Details pertaining to homology modeling of the 3 distinct domains from the proteins FlrC and YfhA, and the results obtained through pair-wise superposition of the corresponding domains from the two different proteins. Click here for file

Additional file 8 **Stochastic state transitions.** Analysis of state transitions obtained by simulating the Boolean model allowing asynchronous updates. Click here for file

Additional file 9 **Boolean model simulation with mutated*yfhA.*** Transition of states obtained through Boolean model simulation with mutated *yfhA*. Click here for file

Additional file 10 **Logical rules for the Boolean model.** Logical rules defined for activation of each of the 34 nodes in the Boolean model. Click here for file
