# Supplementary material for: Understanding the sequential activation of Type III and Type VI Secretion Systems in Salmonella typhimurium using Boolean modeling
Source: Gut Pathog. 2013 Sep 30;5:28. doi: 10.1186/1757-4749-5-28 (PMC3849742; doi:10.1186/1757-4749-5-28)
Supplement: Additional file 1 — Components of the gene regulatory network. List of the genes (and RNAs) included in the network representing the cross-talk among regulatory elements of Type III and Type VI Secretion Systems in Salmonella typhimurium. The synonym codes correspond to the strain S. typhimurium LT2. [file 1757-4749-5-28-S1.pdf]

Additional file 1

List of the genes (and RNAs) included in the network representing the cross-talk among regulatory elements of Type III and Type VI secretion systems in *Salmonella typhimurium*. The synonym codes correspond to the strain *S. typhimurium* LT2.

Genes	Synonym code	Annotation	Comments
mlc	STM1488	Pts operon transcriptional repressor	NCBI annotation
hilD	STM2875	invasion protein regulatory protein	
hilC	STM2867	invasion regulatory protein	
rtsA	STM4315	DNA-binding protein	
hilA	STM2876	invasion protein regulator	
ihf	STM1339 (ihfA), STM0982 (ihfB)	Integration host factor subunit alpha and beta	
sirA	STM1947	Response regulator	
barA	STM2958	Hybrid sensory histidine kinase	
csrA	STM2826	Carbon storage regulator	
fur	STM0693	Ferric uptake regulator	
hns	STM1751	Global DNA-binding transcriptional global regulator	
phoP	STM1231	DNA-binding transcriptional regulator	
slyA	STM1444	Transcriptional regulator	
ssrA	STM1392	Sensor kinase	
ssrB	STM1391	Transcriptional activator	
fis	STM3385	DNA-binding protein	
envZ	STM3501	Osmolarity sensor protein	
ompR	STM3502	Osmolarity response regulator	
yfhA	STM2562	Transcriptional regulator	
mviA	STM1753	Transcriptional regulator of RpoS	
rcsB	STM2270	Transcriptional regulator	
pmrA	STM4292	DNA-binding transcriptional regulator	
sciS	STM0285	Inner membrane protein	
sciG	STM0272	Chaperone ATPase	
*hilE**	STM4509.S	Transcriptional repressor	Baxter *et al.* 2003
*csrB**	-	Non-coding RNA	Teplitski *et al*. 2003
*csrC**	-	Non-coding RNA	Fortune *et al*. 2006
*vrgS**	STM0289	Homolog of effector protein VrgG	Mulder *et al*. 2012

*Genes encoding 'hypothetical' proteins according to NCBI (National Centre for Biotechnology Information) database or genes producing non-coding RNA. Annotations for these proteins were acquired from the corresponding references mentioned under 'Comments' column in the table.

References:

1. Baxter MA, Fahlen TF, Wilson RL, Jones BD: **HilE interacts with HilD and negatively regulates hilA transcription and expression of the Salmonella enterica serovar Typhimurium invasive phenotype**. *Infect Immun* 2003, **71**:1295–1305.

2. Teplitski M, Goodier RI, Ahmer BMM: **Pathways leading from BarA/SirA to motility and virulence gene expression in Salmonella**. *J Bacteriol* 2003, **185**:7257–7265.

3. Fortune DR, Suyemoto M, Altier C: **Identification of CsrC and Characterization of Its Role in Epithelial Cell Invasion in Salmonella enterica Serovar Typhimurium**. *Infect Immun* 2006, **74**:331–339.

4. Mulder DT, Cooper CA, Coombes BK: **Type VI Secretion System-Associated Gene Clusters Contribute to Pathogenesis of Salmonella enterica Serovar Typhimurium**. *Infect Immun* 2012, **80**:1996–2007.
