# Supplementary material for: Understanding the sequential activation of Type III and Type VI Secretion Systems in Salmonella typhimurium using Boolean modeling
Source: Gut Pathog. 2013 Sep 30;5:28. doi: 10.1186/1757-4749-5-28 (PMC3849742; doi:10.1186/1757-4749-5-28)
Supplement: Additional file 2 — YfhA binding sites. YfhA binding sites found in the upstream region and in the ORF of rcsB. YfhA is known to recognize an 18 base pair long motif [TGTCN(10)GACA]. The results given below indicate the position of the probable binding sites (with respect to the transcription start site), and the number of mismatches allowed while considering a hit. [file 1757-4749-5-28-S2.pdf]

Additional file 2

YfhA binding sites found in the upstream region and in the ORF of *rcsB*. YfhA is known to recognize an 18 base pair long motif [TGTCN(10)GACA]. The results given below indicate the position of the probable binding sites (with respect to the transcription start site), and the number of mismatches allowed while considering a hit.

Query region	Length of ORF/upstream region (bp)	Position of probable binding site	Mismatches
Upstream region of the gene *rcsB*	2686	-2304..-2285	1
		-668..-651<br>-1155..-1138<br>-1755..-1738<br>-2033..-2016<br>-2093..-2076<br>-2349..-2332	2
Upstream region of the operon containing *rcsB*<br><br>(position of probable binding sites are given with respect to the transcription start site of the first gene in the operon)	3113	-1665..-1648<br>-2482..-2465	1
		-97..-80<br>-746..-729<br>-1052..-1035<br>-1333..-1316<br>-1399..-1382<br>-1565..-1548<br>-1745..-1728<br>-2316..-2299<br>-2547..-2530	2
ORF of *rcsB*	652	93..110<br>170..187<br>401..418<br>403..420	2
