# Supplementary material for: Understanding the sequential activation of Type III and Type VI Secretion Systems in Salmonella typhimurium using Boolean modeling
Source: Gut Pathog. 2013 Sep 30;5:28. doi: 10.1186/1757-4749-5-28 (PMC3849742; doi:10.1186/1757-4749-5-28)
Supplement: Additional file 3 — Transcription factor binding sites predicted by Tfsitescan. Transcription factor binding sites predicted by "Tfsitescan" in the upstream regions of Type VI Secretion System genes (sciS, vrgS and sciG) and the genes of their regulators (rcsB and pmrA). [file 1757-4749-5-28-S3.pdf]

Additional file 3

Transcription factor binding sites predicted by "Tfsitescan" in the upstream regions of Type VI secretion system genes (*sciS*, *vrgS* and *sciG*) and the genes of their regulators (*rcsB* and *pmrA*). [In cases where genes were preceded by other genes in their respective transcription units (operons), the search was performed in the upstream regions of the corresponding operons]

Gene	Length of upstream region (bp)	Transcriptional regulator	Position of probable binding site	Known function of transcriptional regulator
sciS	202	FlrC	-180 .. -175	Involved in flagellar biogenesis
		DcuR	-149 .. -144	Involved in C4-dicarboxylate metabolism
vrgS	397	DcuR	-392 .. -387	Involved in C4-dicarboxylate metabolism
			-277 .. -272	
			-262 .. -257	
			-232 .. -227	
			-226 .. -221	
			-148 .. -143	
			-99 .. -94	
			-98 .. -93	
			-97 .. -92	
			-73 .. -68	
			-35 .. -30	
			-31 .. -26	
			-30 .. -25	
			-20 .. -15	
			-19 .. -14	
		MalT	-384 .. -379	Transcriptional activator of the genes involved in maltose transport and degradation
		NarL-NarP (present in S. typhimurium)	-326 .. -320	Response regulators involved in anaerobic respiration
		ToxT	-272 .. -267	AraC family transcriptional activator involved in virulence gene expression in *V. cholerae*
		ExsA	-230 .. -223	AraC family regulator involved in transcription of T3SS regulon in *Pseudomonas aeruginosa*
			-152 .. -145	
			-146 .. -139	
			-33 .. -26	
		TraA	-227 .. -221	Involved in transfer of antibiotic genes in human colonic Bacteroides species
			-148 .. -142	
			-35 .. -29	

		CynR	-199 .. -191	Involved in positive regulation of cyn operon (involved in detoxification of exogenous cyanate) in *E. coli*.
		NagC	-195 .. -189	Transcriptional regulator of PTS gene, which facilitates folate biosynthasis
		CytR-half-site	-80 .. -73	Transcriptional repressor involved in nucleoside and deoxynucleoside uptake and metabolism
		TATA box	-75 .. -70	RNA polymerase binding site in archaea an eukaryotes
sciG	368	DcuR	-368 .. -363	Involved in C4-dicarboxylate metabolism
			-367 .. -362	
			-311 .. -306	
			-203 .. -198	
		NarL-NarP	-363 .. -357	Response regulators involved in anaerobic respiration
rcsB	3113	NifA	-3085 .. -3080	Transcriptional activator of Nif (nitrogen fixation) gene
			-3058 .. -3053	
			-2373 .. -2368	
			-2337 .. -2332	
		DcuR	-3034 .. -3029	Involved in C4-dicarboxylate metabolism
			-3033 .. -3028	
			-3032 .. -3027	
			-3005 .. -3000	
			-2982 .. -2977	
			-2981 .. -2976	
			-2242 .. -2237	
			-2216 .. -2211	
			-1929 .. -1924	
			-1865 .. -1860	
			-1592 .. -1587	
			-1577 .. -1572	
			-914 .. -909	
			-821 .. -816	
			-809 .. -804	
			-804 .. -799	
			-726 .. -721	
			-704 .. -699	
			-604 .. -599	
			-595 .. -590	
			-563 .. -558	
			-537 .. -532	

			-536 .. -531	
			-479 .. -474	
			-478 .. -473	
			-477 .. -472	
			-463 .. -458	
			-411 .. -406	
			-380 .. -375	
			-376 .. -371	
		MalT	-2637 .. -2632	Transcriptional activator of the genes involved in maltose transport and degradation
		RbtR	-2577 .. -2570	A repressor of RbtR operon involved in pentitol metabolism in *Klebsiella aerogenes*
		Nitrogen regulation	-2497 .. -2491	
rcsB	3113	NifA	-2494 .. -2488	Transcriptional activator of Nif (nitrogen fixation) gene
		FlrC	-2433 .. -2428	Involved in flagellar biogenesis
			-1999 .. -1994	
			-817 .. -812	
			-654 .. -649	
		NarL-NarP	-2213 .. -2207	Response regulators involved in anaerobic respiration
			-2032 .. -2026	
			-1535 .. -1529	
			-861 .. -855	
			-343 .. -337	
		ToxT	-1943 .. -1938	AraC family transcriptional activator involved in virulence gene expression in *V. cholerae*
		ExsA	-1908 .. -1901	AraC family regulator involved in transcription of T3SS regulon in *Pseudomonas aeruginosa*
			-1272 .. -1265	
			-562 .. -555	
			-539 .. -532	
			-518 .. -511	
			-309 .. -302	
		GalR	-1450 .. -1443	Transcriptional repressor of gal operon
		PhoP	-1449 .. -1444	
		TATA box	-806 .. -801	RNA polymerase binding site in archaea an eukaryotes
		OmpR-OmpC	-612 .. -577	OmpR is a response regulator of the outer membrane protein OmpC, involved in export of toxic salt

		NagC-half-site	-515 .. -509	Transcriptional regulator of PTS gene, which facilitates folate biosynthasis
		MalI	-434 .. -427	Involved in regulation of maltose system in *E. coli*
		TraA	-380 .. -374	Involved in transfer of antibiotic genes in human colonic Bacteroides species
pmrA	166	RbtR	-2577 .. -2570	A repressor of RbtR operon involved in pentitol metabolism in *Klebsiella aerogenes*
		MalT	-124 .. -119	Transcriptional activator of the genes involved in maltose transport and degradation
			-110 .. -105	
		DcuR	-84 .. -79	Involved in C4-dicarboxylate metabolism
			-82 .. -77	
			-13 .. -8	
			-12 .. -7	
		CRP-ara	-78 .. -68	cAMP receptor protein involved in regulation of gal operon
		MalI	-39 .. -32	Involved in regulation of maltose system in *E. coli*
		NifA	-27 .. -22	Transcriptional activator of Nif (nitrogen fixation) gene

Note: Apart from FlrC (discussed in detail in the Results and Discussion section), several other transcription factor binding sites were detected. Out of these, the transcription factors which were earlier observed to be directly involved in virulence are ToxT and ExsA. Search for homologs of ToxT in *S. typhimurium* returned a gene named AdiY, which was known to be involved in Salmonella replication inside the macrophage (Choi *et al.*, 2012). However, since the mechanism of action of this protein was completely unknown, ToxT was not considered for the Boolean model. ExsA was reported to be a transcriptional activator of T3SS in *Pseudomonas aeruginosa* (Brutinel *et al.*, 2009) and a search for its homolog in *S. typhimurium* matched a protein named STM1082. However, since T3SS of Salmonella is already well studied and well represented in our network, we refrained from adding a new entity in the T3SS sub-network, without being sure of its mode interaction (if any). Other transcription factors (for which binding sites were predicted) included DcuR, involved in C4-dicarboxylate metabolism, NifA, involved in activation of nitrogen fixation genes in

diazotrophic bacteria, MalT, involved in regulation of genes for malto-oligosaccharide utilization, etc.

References:

1. Choi Y, Choi J, Groisman EA, Kang D-H, Shin D, Ryu S: **Expression of STM4467-encoded arginine deiminase controlled by the STM4463 regulator contributes to Salmonella enterica serovar Typhimurium virulence**. *Infect Immun* 2012, **80**:4291–4297.

2. Brutinel ED, Vakulskas CA, Yahr TL: **ExsD Inhibits Expression of the Pseudomonas aeruginosa Type III Secretion System by Disrupting ExsA Self-Association and DNA Binding Activity**. *J Bacteriol* 2010, **192**:1479–1486.
