# Supplementary material for: Understanding the sequential activation of Type III and Type VI Secretion Systems in Salmonella typhimurium using Boolean modeling
Source: Gut Pathog. 2013 Sep 30;5:28. doi: 10.1186/1757-4749-5-28 (PMC3849742; doi:10.1186/1757-4749-5-28)
Supplement: Additional file 4 — FlrC binding sites. FlrC-binding sites predicted through 'Tfsitescan’ in the upstream regions and within the ORFs of sciS and rcsB. [file 1757-4749-5-28-S4.pdf]

Additional file 4

FlrC-binding sites predicted through 'Tfsitescan' in the upstream regions and within the ORFs of *sciS* and *rcsB*.

Gene	Query region	Length of ORF/Upstream region (bp)	Position of FlrC binding site (CGGCAA)
sciS	Upstream region	202	-180..-175
	ORF	3870	59..602
			1021..1026
			1090..1095
			1488..1493
			2270..2275
			2828..2833
			2915..2920
rcsB	Upstream region	3113	-654..-649
			-817..-812
			-1999..-1994
			-2433..-2428
	ORF	651	23..28

Note: In cases where the genes were preceded by other genes in their respective transcription units (operons), the search was performed in the upstream regions of the corresponding operons.
