# Supplementary material for: Understanding the sequential activation of Type III and Type VI Secretion Systems in Salmonella typhimurium using Boolean modeling
Source: Gut Pathog. 2013 Sep 30;5:28. doi: 10.1186/1757-4749-5-28 (PMC3849742; doi:10.1186/1757-4749-5-28)
Supplement: Additional file 5 — Homology models of FlrC and YfhA. Details pertaining to homology modeling of the proteins FlrC and YfhA and results of superposition of the two modelled structures. [file 1757-4749-5-28-S5.pdf]

Additional file 5
Homology modeling of FlrC

Template PDB structure:	1ny5 (2.40 angstroms)
Query sequence length:	479
Modelled residue range:	7 – 375
Sequence Identity with template:	39.79%
E-value:	0.00e-1

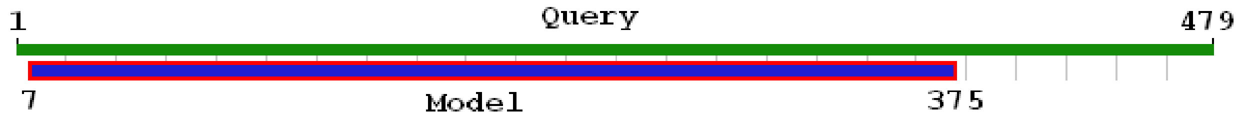

Model quality:

QMEAN4 score *	= 0.66
QMEAN Z-score	= - 1.88

[* *Benkert P, Biasini M, Schwede T. (2011). "Toward the estimation of the absolute quality of individual protein structure models." Bioinformatics, 27(3):343-50.*]

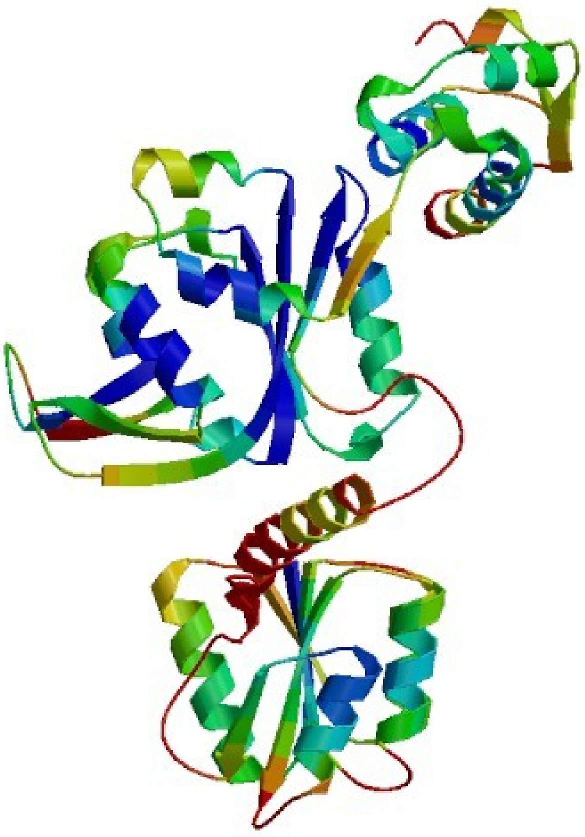

Supporting Figure S1. Homology model of FlrC with reliability of the model structure represented as a colour gradient from blue to red. While the blue colour signifies better reliability, the red colour represents potentially unreliable regions. [Figure generated with Swiss-Model]

Homology modeling of YfhA

Template PDB structure:	1ny5 (2.40 angstroms)
Query sequence length:	445
Modelled residue range:	7 – 382
Sequence Identity with template:	38.28%
E-value:	0.00e-1

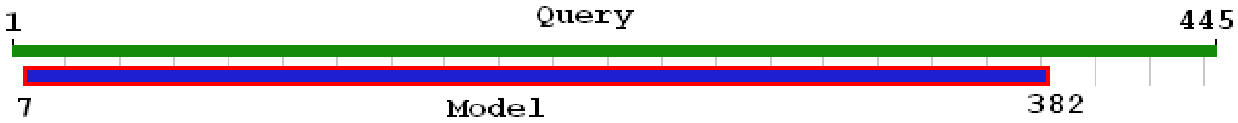

Model quality:

QMEAN4 score *	=	0.81
QMEAN Z-score	=	0.74

[* *Benkert P, Biasini M, Schwede T. (2011). "Toward the estimation of the absolute quality of individual protein structure models." Bioinformatics, 27(3):343-50.*]

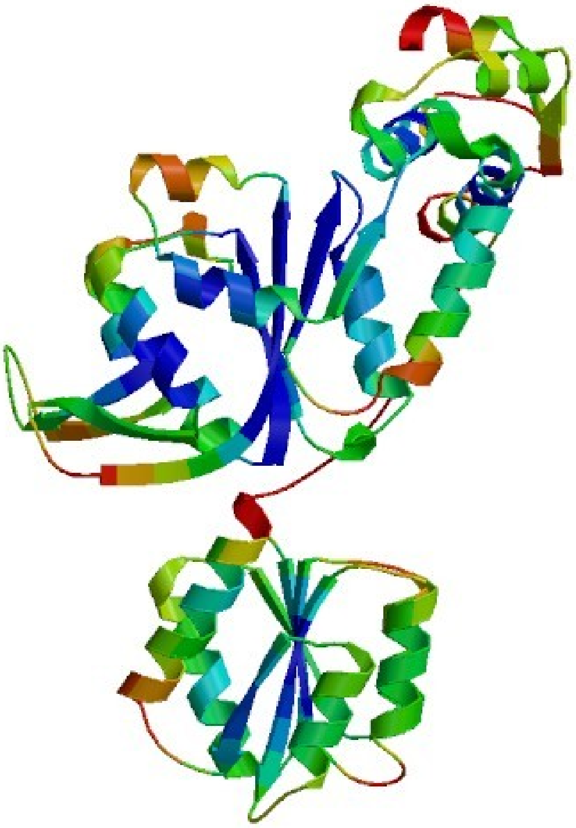

Supporting Figure S2. Homology model of YfhA with reliability of the model structure represented as a colour gradient from blue to red. While the blue colour signifies better reliability, the red colour represents potentially unreliable regions. [Figure generated with Swiss-Model]

Superposition of Homology Models

The homology models of FlrC and YfhA were superposed using the 'FAST Alignment and Search Tool' (Zhu and Weng 2005), the result of which is given below-

```
FAST ALIGNMENT: FlrC.pdb YfhA.pdb
L=347 SX=1.052e+04 SN=2.823e+01 L1=369 L2=376 RMSD=1.325

 1:    -KVLIVEDDEGLREALIDTLALAGYEWLEADCAEDALLKLKSHSVDIVVSDVQMAGMGGL
 2:    AHLLLVDDDPGLLKLLGMRLTSEGYSVVTAESGQEGLRVLHREKVDLVISDLRMDEMDGM

 1:    ALLRSIKQHWPNLPVLLMTAYANIQDAVSAMKDGAIDYMAKPFAPEV---LLNMVSRYAP
 2:    QLFTEIQKVQPGMPVIILTAHGSIPDAVAATQKGVFSFLTKPIDR--DALYKAIDEALEQ

 1:    VKSDDNGDAVVADTKSLKLLALAD------------------------KVAKTDANVMIL
 2:    SAPA--------------------TDDSWRNAIVTRSPLMLRLLEQARMVAQSDVSVLIN

 1:    GPSGSGKEVMSRYIHNASPRKEGPFIAINCAAIPDNMLEATLFGYEKGAFTGAVQACPGK
 2:    GQSGTGKEIFAQAIHNASPRSNKPFVAINCGALPEQLLESELFGHARGAFTGAVSNREGL

 1:    FEQAQGGTILLDEISEMDLNLQAKLLRVLQEREVERLGSRKSIKLDVRVLATSNRDLKQY
 2:    FQAAEGGTLFLDEIGDMPAPLQVKLLRVLQERKVRPLGSNRDIDIDVRIISATHRDLPKA

 1:    VQAGHFREDLYYRLNVFPLTWPALCERKDDIEPLANHLIERHCKKLGLPVPSIAPNAITK
 2:    MARGEFREDLYYRLNVVSLKIPALAERTEDIPLLANHLLRQSAQRHKPFVRAFSTDAMKR

 1:    LLNYPWPGNVRELDNVVQRALILSENGHIQSEHILLE-*
 2:    LMTASWPGNVRQLVNVIEQCVALTSSPVISDALVEQAL*
```

Superposed structure:

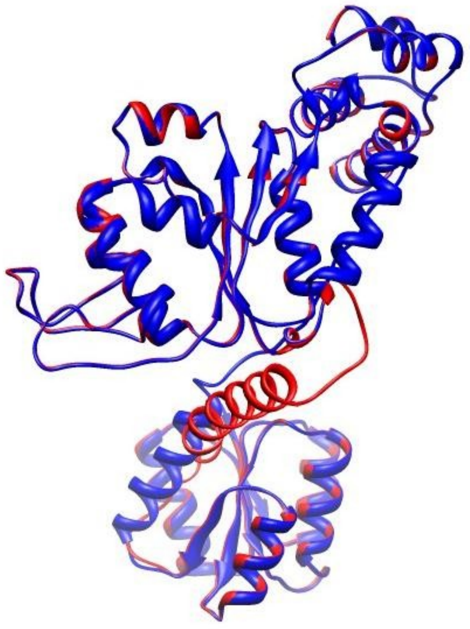

Supporting Figure S3. Structures of FlrC and YfhA, as predicted by homology modeling, are superposed on each other.

The structures of FlrC and YfhA have been represented in red and blue respectively.

The RMSD between the two predicted structures were calculated to be 1.325 angstroms.
