# Supplementary material for: Understanding the sequential activation of Type III and Type VI Secretion Systems in Salmonella typhimurium using Boolean modeling
Source: Gut Pathog. 2013 Sep 30;5:28. doi: 10.1186/1757-4749-5-28 (PMC3849742; doi:10.1186/1757-4749-5-28)
Supplement: Additional file 6 — Pfam Domains identified in FlrC and YfhA. [file 1757-4749-5-28-S6.pdf]

Additional file 6

Domains identified in FlrC and YfhA using 'Pfam' search (http://pfam.janelia.org/search).

Protein	Domain position	Family	Description
FlrC	8-117	Response_reg	Response regulator receiver domain
	137-297	Sigma54_activat	Sigma-54 interaction domain
	430-470	HTH_8	Bacterial regulatory protein, Fis family
YfhA	9-118	Response_reg	Response regulator receiver domain
	137-303	Sigma54_activat	Sigma-54 interaction domain
	398-430	HTH_8	Bacterial regulatory protein, Fis family
