# Supplementary material for: Understanding the sequential activation of Type III and Type VI Secretion Systems in Salmonella typhimurium using Boolean modeling
Source: Gut Pathog. 2013 Sep 30;5:28. doi: 10.1186/1757-4749-5-28 (PMC3849742; doi:10.1186/1757-4749-5-28)
Supplement: Additional file 7 — Homology modeling of three domains of FlrC and YfhA. Details pertaining to homology modeling of the 3 distinct domains from the proteins FlrC and YfhA, and the results obtained through pair-wise superposition of the corresponding domains from the two different proteins. [file 1757-4749-5-28-S7.pdf]

Additional file 7

Homology modeling of 3 domains of FlrC and YfhA proteins

Details of homology modeling

Protein	Domain/Segment	Query Sequence Length	Template (PDB ID)	Sequence Identity with template	E-value	Modelled Residue range	QMEAN4 score *	QMEAN Z-score *
FlrC	Response regulator receiver domain	110	1l5yB (2.10 A)	42.73%	4.30E-29	1-110	0.787	-0.211
	Sigma-54 interaction domain	161	1ny5B (2.40 A)	51.88%	0.00E+00	2-161	0.862	0.777
	DNA-binding domain (HTH)	41	1ojlD (3.00 A)	40.00%	2.60E-11	1-40	0.842	1.123
YfhA	Response regulator receiver domain	110	1l5yB (2.10 A)	39.09%	4.60E-28	1-110	0.811	0.023
	Sigma-54 interaction domain	167	1ojlD (3.00 A)	59.28%	0.00E+00	1-167	0.667	-1.512
	DNA-binding domain (HTH)	33	3jrhB (2.88 A)	39.39%	3.60E-05	1-33	0.849	1.152

[* *Benkert P, Biasini M, Schwede T. (2011). "Toward the estimation of the absolute quality of individual protein structure models." Bioinformatics, 27(3):343-50.*]

Note: The modeled structures are depicted in Figure 3 of the article.

Superposition of Homology Models

Pair-wise superposition of REC, AAA, and HTH domains of FlrC and YfhA proteins was done using the 'FAST Alignment and Search Tool' (Zhu and Weng 2005), the results of which are given below-

ALIGNMENT OF RECEIVER (REC) DOMAINS:

```
FAST ALIGNMENT: FlrC_Response_reg_8-117.pdb YfhA_Response_reg_9-118.pdb
L=110 SX=3.043e+03 SN=2.766e+01 L1=110 L2=110 RMSD=0.086

 1:    VLIVEDDEGLREALIDTLALAGYEWLEADCAEDALLKLKSHSVDIVVSDVQMAGMGGLAL
 2:    LLLVDDDPGLLKLLGMRLTSEGYSVVTAESGQEGLRVLHREKVDLVISDLRMDEMDGMQL

 1:    LRSIKQHWPNLPVLLMTAYANIQDAVSAMKDGAIDYMAKPFAPEVLLNMV*
 2:    FTEIQKVQPGMPVIILTAHGSIPDAVAATQKGVFSFLTKPIDRDALYKAI*
```

ALIGNMENT OF SIGMA-54 INTERACTION DOMAINs (AAA):

```
FAST ALIGNMENT: FlrC_Sigma54_activat_137-297.pdb YfhA_Sigma54_activat_137-303.pdb
L=159 SX=3.579e+03 SN=2.190e+01 L1=160 L2=167 RMSD=1.468

 1:    -------SLKLLALADKVAKTDANVMILGPSGSGKEVMSRYIHNASPRKEGPFIAINCAA
 2:    IVTRSPLMLRLLEQARMVAQSDVSVLINGQSGTGKEIFAQAIHNASPRSNKPFVAINCGA

 1:    IPDNMLEATLFGYEKGA-FTGAVQACPGKFEQAQGGTILLDEISEMDLNLQAKLLRVLQE
 2:    LPEQLLESELFGHARGAFTGAV-SNREGLFQAAEGGTLFLDEIGDMPAPLQVKLLRVLQE

 1:    REVERLGSRKSIKLDVRVLATSNRDLKQYVQAGHFREDLYYRLNVFPL*
 2:    RKVRPLGSNRDIDIDVRIISATHRDLPKAMARGEFREDLYYRLNVVSL*
```

ALIGNMENT OF DNA BINDING (HTH) DOMAINS:

```
FAST ALIGNMENT: FlrC_HTH_8_430-470.pdb YfhA_HTH_8_398-430.pdb
L=32 SX=2.794e+02 SN=7.687e+00 L1=40 L2=33 RMSD=0.789

 1:    LRD-QEFAIILDTLAECQGRRKEMAEKLGISPRTLRYKLAK*
 2:    ---QFELNYLRKLLQITKGNVTHAARMAGRNRTEFY-----*
```
