# Supplementary material for: Understanding the sequential activation of Type III and Type VI Secretion Systems in Salmonella typhimurium using Boolean modeling
Source: Gut Pathog. 2013 Sep 30;5:28. doi: 10.1186/1757-4749-5-28 (PMC3849742; doi:10.1186/1757-4749-5-28)
Supplement: Additional file 8 — Stochastic state transitions. Analysis of state transitions obtained by simulating the Boolean model allowing asynchronous updates. [file 1757-4749-5-28-S8.pdf]

Additional File 8

Sequential activation of SPI1 → SPI2 → T6SS

The Boolean model presented in this work has been simulated under two different conditions, viz., distal-intestinal lumen environment and intra-macrophageal environment, which are encountered by the invading Salmonella cells in subsequent stages of infection. The simulations have been run using both the synchronous and asynchronous update schemes. Adopting the asynchronous update scheme allows updation of any randomly selected node at a time step, thereby making the outcomes stochastic. In case there are more than one possible steady-states/attractors for the system, it is possible to reach each of these attractors using an asynchronous update scheme (through a large number of replicate simulations), even if the initial condition remains the same.

When simulated under the first condition (distal-intestinal lumen environment - as mentioned in Table 1A), using either the synchronous or asynchronous update schemes, the simulation converges to a single attractor/stable-state – having SPI1 turned 'on' and the other two secretion systems turned 'off'. Running simulations for the intra-macrophageal environment also results in a single stable state with the T6SS turned on, and the other two secretion systems turned 'off'. The state transitions observed using the synchronous update scheme (Table 1B) suggests a sequential activation of SPI1 → SPI2 → T6SS, i.e., the system traverses through an intermediate step, when SPI2 is turned 'on', before encountering any step where T6SS is activated.

In addition, the state transitions obtained with the asynchronous update scheme were further analyzed to investigate the several possible routes leading to the T6SS activation (Figure S1). All the possible states of the system were grouped according to the conditions of SPI1, SPI2 and T6SS. For example, all the states of the system having SPI1 turned 'on' and with SPI2 and T6SS in an inactivated state would be members of the group '100'. These (groups of) states have been indicated in Figure S1 as individual nodes. The transition probabilities from each of these states (nodes) to any other were calculated from the results of the asynchronous simulation(s). Consequently, the probabilities of all possible paths, starting from the node '100' (representing the initial state for the intra-macrophageal simulation) to '001' (the stable state) were calculated. It was observed that the combined probabilities of the paths indicating sequential activation was at least ~66% compared to a ~20% chance that the system would traverse through paths not leading to sequential activation. If the feedback loops in the state transition graph are also considered, the probability of observing sequential activation is as high as ~76%.

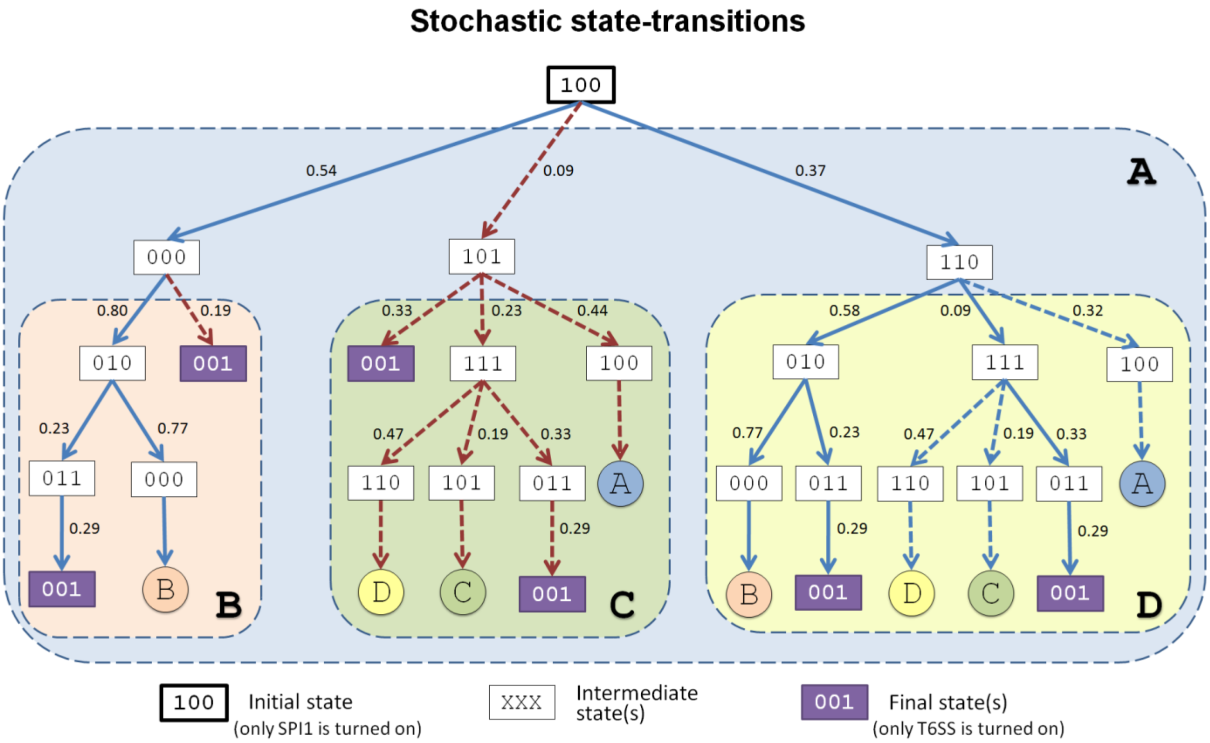

Each node in the graph represents different states through which the modeled Boolean network traverses during an asynchronous simulation (mimicking the intra-macrophageal environment). The 3 digits depict the states of the 3 secretion systems - SPI1, SPI2, and T6SS respectively. The edges (arrows) represent transitions from one state to another (transition probabilities have also been indicated). For easy depiction of feedback loops (present in some of the paths), 4 sub-graphs (A, B, C and D) have been indicated.

Sequential activation of SPI1 → SPI2 → T6SS are represented by paths which traverse through intermediate state(s) with SPI2 turned 'on' before T6SS is activated. Some of the paths containing feedback loops may partially lead to sequential activation, i.e., only a fraction of paths downstream of the loop will lead to the necessary intermediate steps.

→ (solid blue)	Paths leading to sequential activation	100 → 000 → 010 → XXX<br>100 → 110 → 010 → XXX<br>100 → 110 → 111 → 011 → XXX	0.44<br>0.21<br>0.01
- - -→ (dashed red)	Paths not leading to sequential activation	100 → 000 → 001<br>100 → 101 → XXX	0.11<br>0.09
- - -→ (dashed blue)	Paths partially leading to sequential activation		

probabilities

Figure S1. Possible paths/routes leading to the T6SS activation
