# Supplementary material for: Understanding the sequential activation of Type III and Type VI Secretion Systems in Salmonella typhimurium using Boolean modeling
Source: Gut Pathog. 2013 Sep 30;5:28. doi: 10.1186/1757-4749-5-28 (PMC3849742; doi:10.1186/1757-4749-5-28)
Supplement: Additional file 9 — Boolean model simulation with mutatedyfhA. Transition of states obtained through Boolean model simulation with mutated yfhA. [file 1757-4749-5-28-S9.pdf]

Additional file 9

State transitions obtained through simulating the Boolean model with a 'mutated' *yfhA*. The initial states of the environmental factors were set according to their availability inside macrophage. The rows represent the dynamic behavior of the network (constituting 34 nodes) under study. '0' represents inactive state and '1' represents active state. Row 1 represents the values specified as the initial states for simulation. The dark-gray cells correspond to a node's transit from an inactive state to an active state (0 → 1) or vice-versa (1 → 0). The model reaches a stable state in the 5th step, but fails to achieve a state where T6SS is activated.

	Nodes																																	
State Transition	**Glucose**	**Iron**	**Magnesium**	**Calcium**	**Osmolarity**	**Stationary phase**	Mlc	HilE	HilD	HilC	RtsA	HilA	Ihf	SirA-BarA	CsrBC	CsrA	Fur	H-NS	PhoP	SlyA	SsrAB	Fis	EnvZ	OmpR	YfhA	MviA	RcsB	PmrA	SciS	VrgS	SciG	**SPI -1**	**SPI -2**	**T6SS**
1	1	1	0	0	0	1	0	0	1	1	1	1	0	0	0	0	0	0	0	0	0	0	0	0	0	0	0	0	0	0	0	1	0	0
2	1	1	0	0	0	1	0	**1**	1	1	1	1	0	0	0	**1**	**1**	**1**	**1**	**1**	0	**1**	0	**1**	0	0	0	0	0	0	0	1	0	0
3	1	1	0	0	0	1	0	1	**0**	1	1	**0**	0	0	0	1	1	1	1	1	**1**	1	0	1	0	0	0	**1**	0	0	0	1	0	0
4	1	1	0	0	0	1	0	1	0	1	1	0	0	0	0	1	1	1	1	1	**0**	1	0	1	0	0	0	1	0	**1**	0	**0**	**1**	0
5	1	1	0	0	0	1	0	1	0	1	1	0	0	0	0	1	1	1	1	1	0	1	0	1	0	0	0	1	0	1	0	0	**0**	0
