# Supplementary material for: Understanding the sequential activation of Type III and Type VI Secretion Systems in Salmonella typhimurium using Boolean modeling
Source: Gut Pathog. 2013 Sep 30;5:28. doi: 10.1186/1757-4749-5-28 (PMC3849742; doi:10.1186/1757-4749-5-28)
Supplement: Additional file 10 — Logical rules for the Boolean model. Logical rules defined for activation of each of the 34 nodes in the Boolean model. [file 1757-4749-5-28-S10.pdf]

Additional file 10

Logical rules for activation of each of the 34 nodes in the Boolean model.

Node	Logical rule	References
Glucose	Input node	Bowden *et al*. 2009
Iron	Input node	Teixido *et al*. 2011, Kortman *et al*. 2012
Magnesium	Input node	Miao and Miller 2000
Calcium	Input node	Linehan *et al*. 2005
Osmolarity	Input node	Lee *et al*. 2000, Linehan *et al*. 2005
Stationary phase	Input node	Ibarra *et al*. 2010
Mlc	NOT Glucose	Lim *et al*. 2007
HilE	NOT Mlc	Lim *et al*. 2007
HilD	SirA-BarA OR HilD OR HilC OR RtsA OR Fur AND (NOT CsrA) AND (NOT HilE)	Baxter *et al*. 2003, Lawhon *et al*. 2003, Ellermeier *et al*. 2005, Ellermeier *et al*. 2008, Mizusaki *et al*. 2008, Ganesh *et al*. 2009, Saini *et al*. 2010, Teixido *et al*. 2011
HilC	HilD OR HilC OR RtsA	Ellermeier *et al*. 2005, Saini *et al*. 2010
RtsA	HilD OR HilC OR RtsA	Ellermeier *et al*. 2005, Saini *et al*. 2010
HilA	HilD AND (HilC OR RtsA) AND (NOT PhoP) AND ((NOT H-NS) OR (H-NS AND IHF) OR SirA)	Boddicker *et al*. 2003, Schechter *et al*. 2003, Ellermeier *et al*. 2005, Aguirre *et al*. 2006, Saini *et al*. 2010, Queiroz *et al*. 2011
IHF	Osmolarity AND Stationary phase	Queiroz *et al*. 2011
SirA-BarA	Osmolarity AND (NOT Glucose)	Teplitski *et al*. 2003, Lim *et al*. 2007, Mizusaki *et al*. 2008
CsrBC	SirA-BirA	Teplitski *et al*. 2003, Fortune *et al*. 2006
CsrA	NOT CsrBC	Fortune *et al*. 2006
Fur	Iron	Teixido *et al*. 2011, Troxell *et al*. 2011
H-NS	NOT (PhoP AND Fur AND SlyA AND HilD AND IHF)	Bustamante *et al*. 2008, Perez *et al*. 2008, Troxell *et al*. 2011
PhoP	NOT Magnesium	Bijlsma *et al*. 2005
SlyA	(NOT Osmolarity) AND (NOT Calcium)	Linehan *et al*. 2005
SsrAB	OmpR AND (((NOT H-NS) AND (HilD OR SlyA OR PhoP)) OR (H-NS AND (HilD OR (PhoP AND SlyA))))	Lee *et al*. 2000, Bijlsma *et al*. 2005, Linehan *et al*. 2005, Bustamante *et al*. 2008, Martinez *et al*. 2011

Fis	Stationary phase	Ibarra *et al*. 2010
EnvZ	Osmolarity	Lee *et al*. 2000
OmpR	NOT EnvZ	Feng *et al*. 2003
YfhA	NOT EnvZ	Yamamoto *et al*. 2005
MviA	H-NS AND (NOT PhoP)	Garcia-Calderon *et al*. 2009
RcsB	YfhA AND (NOT MviA)	David Ghosh 2000, Garcia-Calderon *et al*. 2009
PmrA	PhoP	Soncini and Groisman 1996
SciS	(RcsB AND PmrA) OR (RcsB AND PmrA AND YfhA) AND (NOT SsrAB)	David Ghosh 2000, Parsons and Heffron 2005, Wang *et al*. 2011, Leung *et al*. 2011
VrgS	PmrA	Wang *et al*. 2011
SciG	RcsB AND PmrA	Wang *et al*. 2011
SPI-1	HilA	Ellermeier *et al*. 2005
SPI-2	SsrAB AND (SlyA or Fis)	Cirillo *et al*. 1998, Linehan *et al*. 2005, Lim *et al.* 2006
TSSS	SciS AND SciG AND VrgS	Wang *et al*. 2011

REFERENCES

Aguirre A, Cabeza ML, Spinelli SV, McClelland M, García Véscovi E, Soncini FC: **PhoP-induced genes within Salmonella pathogenicity island 1**. *J. Bacteriol*. 2006, **188:**6889–68

Baxter MA, Fahlen TF, Wilson RL, Jones BD: **HilE Interacts with HilD and Negatively Regulates hilA Transcription and Expression of the Salmonella enterica Serovar Typhimurium Invasive Phenotype**. *Infect Immun* 2003, **71**:1295–1305.

Boddicker JD, Knosp BM, Jones BD: **Transcription of the Salmonella invasion gene activator, hilA, requires HilD activation in the absence of negative regulators**. *J Bacteriol* 2003, **185**:525–533.

Bijlsma JJE, Groisman EA: **The PhoP/PhoQ system controls the intramacrophage type three secretion system of Salmonella enterica**. *Mol Microbiol* 2005, **57**:85–96.

Bustamante VH, Martínez LC, Santana FJ, Knodler LA, Steele-Mortimer O, Puente JL: **HilD-mediated transcriptional cross-talk between SPI-1 and SPI-2**. *Proc Natl Acad Sci USA* 2008, **105**:14591–14596.

Bowden SD, Rowley G, Hinton JCD, Thompson A: **Glucose and glycolysis are required for the successful infection of macrophages and mice by Salmonella enterica serovar typhimurium**. *Infect Immun* 2009, **77**:3117–3126.

Cirillo DM, Valdivia RH, Monack DM, Falkow S: **Macrophage-dependent induction of the Salmonella pathogenicity island 2 type III secretion system and its role in intracellular survival**. *Mol Microbiol* 1998, **30**:175–188.

Ellermeier CD, Ellermeier JR, Slauch JM: **HilD, HilC and RtsA constitute a feed forward loop that controls expression of the SPI1 type three secretion system regulator hilA in Salmonella enterica serovar Typhimurium**. *Mol Microbiol* 2005, **57**:691–705.

Ellermeier JR, Slauch JM: **Fur regulates expression of the Salmonella pathogenicity island 1 type III secretion system through HilD**. *J Bacteriol* 2008, **190**:476–486.

Feng X, Oropeza R, Kenney LJ: **Dual regulation by phospho-OmpR of ssrA/B gene expression in Salmonella pathogenicity island 2**. *Mol Microbiol* 2003, **48**:1131–1143.

Fortune DR, Suyemoto M, Altier C: **Identification of CsrC and characterization of its role in epithelial cell invasion in Salmonella enterica serovar Typhimurium**. *Infect Immun* 2006, **74**:331–339.

Ghosh D: **Object-oriented Transcription Factors Database (ooTFD)**. *Nucleic Acids Res* 2000, **28**:308–310.

Ganesh AB, Rajasingh H, Mande SS: **Mathematical modeling of regulation of type III secretion system in Salmonella enterica serovar Typhimurium by SirA**. *In Silico Biol (Gedrukt)* 2009, **9**:S57–72.

García-Calderón CB, Casadesús J, Ramos-Morales F: **Regulation of igaA and the Rcs system by the MviA response regulator in Salmonella enterica**. *J Bacteriol* 2009, **191**:2743–2752.

Ibarra JA, Knodler LA, Sturdevant DE, Virtaneva K, Carmody AB, Fischer ER, Porcella SF, Steele-Mortimer O: **Induction of Salmonella pathogenicity island 1 under different growth conditions can affect Salmonella-host cell interactions in vitro**. *Microbiology* 2010, **156**(Pt 4):1120–1133.

Kortman GAM, Boleij A, Swinkels DW, Tjalsma H: **Iron Availability Increases the Pathogenic Potential of Salmonella Typhimurium and Other Enteric Pathogens at the Intestinal Epithelial Interface**. *PLoS ONE* 2012, **7**: e29968.

Lee AK, Detweiler CS, Falkow S: **OmpR regulates the two-component system SsrA-ssrB in Salmonella pathogenicity island 2**. *J Bacteriol* 2000, **182**:771–781.

Lawhon SD, Frye JG, Suyemoto M, Porwollik S, McClelland M, Altier C: **Global regulation by CsrA in Salmonella typhimurium.** *Mol. Microbiol.* 2003, **48**:1633–1645.

Linehan SA, Rytkönen A, Yu X-J, Liu M, Holden DW: **SlyA regulates function of Salmonella pathogenicity island 2 (SPI-2) and expression of SPI-2-associated genes**. *Infect Immun* 2005, **73**:4354–4362.

Lim S, Kim B, Choi H-S, Lee Y, Ryu S: **Fis is required for proper regulation of ssaG expression in Salmonella enterica serovar Typhimurium**. *Microb Pathog* 2006, **41**:33–42.

Lim S, Yun J, Yoon H, Park C, Kim B, Jeon B, Kim D, Ryu S: **Mlc regulation of Salmonella pathogenicity island I gene expression via hilE repression**. *Nucleic Acids Res* 2007, **35**:1822–1832.

Leung KY, Siame BA, Snowball H, Mok Y-K: **Type VI secretion regulation: crosstalk and intracellular communication**. *Curr Opin Microbiol* 2011, **14**:9–15.

Miao EA, Miller SI: **A conserved amino acid sequence directing intracellular type III secretion by Salmonella typhimurium**. *Proc Natl Acad Sci USA* 2000, **97**:7539–7544.

Mizusaki H, Takaya A, Yamamoto T, Aizawa S: **Signal pathway in salt-activated expression of the Salmonella pathogenicity island 1 type III secretion system in Salmonella enterica serovar Typhimurium**. *J Bacteriol* 2008, **190**:4624–4631.

Martínez LC, Yakhnin H, Camacho MI, Georgellis D, Babitzke P, Puente JL, Bustamante VH: **Integration of a complex regulatory cascade involving the SirA/BarA and Csr global regulatory systems that controls expression of the Salmonella SPI-1 and SPI-2 virulence regulons through HilD**. *Mol. Microbiol.* 2011, **80:**1637–1656.

Parsons DA, Heffron F: **sciS, an icmF homolog in Salmonella enterica serovar Typhimurium, limits intracellular replication and decreases virulence**. *Infect Immun* 2005, **73**:4338–4345.

Perez JC, Latifi T, Groisman EA: **Overcoming H-NS-mediated transcriptional silencing of horizontally acquired genes by the PhoP and SlyA proteins in Salmonella enterica**. *J Biol Chem* 2008, **283**:10773–10783.

Queiroz MH, Madrid C, Paytubi S, Balsalobre C, Juárez A: **Integration host factor alleviates H-NS silencing of the Salmonella enterica serovar Typhimurium master regulator of SPI1, hilA**. *Microbiology (Reading, Engl)* 2011, **157**(Pt 9):2504–2514.

Soncini FC, Groisman EA: **Two-component regulatory systems can interact to process multiple environmental signals**. *J Bacteriol* 1996, **178**:6796–6801.

Schechter LM, Jain S, Akbar S, Lee CA: **The small nucleoid-binding proteins H-NS, HU, and Fis affect hilA expression in Salmonella enterica serovar Typhimurium**. *Infect Immun* 2003, **71**:5432–5435.

Saini S, Ellermeier JR, Slauch JM, Rao CV: The **role of coupled positive feedback in the expression of the SPI1 type three secretion system in Salmonella**. *PLoS Pathog* 2010, **6**: e1001025.

Teplitski M, Goodier RI, Ahmer BMM: **Pathways leading from BarA/SirA to motility and virulence gene expression in Salmonella**. *J Bacteriol* 2003, **185**:7257–7265.

Teixidó L, Carrasco B, Alonso JC, Barbé J, Campoy S: **Fur activates the expression of Salmonella enterica pathogenicity island 1 by directly interacting with the hilD operator in vivo and in vitro**. *PLoS ONE* 2011, **6**:e19711.

Troxell B, Sikes ML, Fink RC, Vazquez-Torres A, Jones-Carson J, Hassan HM: **Fur negatively regulates hns and is required for the expression of HilA and virulence in Salmonella enterica serovar Typhimurium**. *J. Bacteriol*. 2011, **193:**497–505.

Wang M, Luo Z, Du H, Xu S, Ni B, Zhang H, Sheng X, Xu H, Huang X: **Molecular characterization of a functional type VI secretion system in Salmonella enterica serovar Typhi**. *Curr Microbiol* 2011, **63**:22–31.

Yamamoto K, Hirao K, Oshima T, Aiba H, Utsumi R, Ishihama A: **Functional characterization in vitro of all two-component signal transduction systems from Escherichia coli**. *J Biol Chem* 2005, **280**:1448–1456.
